# Candling Analysis of Egg Development in an Endangered Bird Species Crested Ibis ( *Nipponia nippon* )

**DOI:** 10.1002/ece3.73797

**Published:** 2026-06-16

**Authors:** Yuansi He, Xuebo Xi, Siyi Zeng, Hua Huang, Xiangjiang Zhan, Daiping Wang

**Affiliations:** ^1^ State Key Laboratory of Animal Biodiversity Conservation and Integrated Pest Management, Institute of Zoology Chinese Academy of Sciences Beijing China; ^2^ University of Chinese Academy of Sciences Beijing China; ^3^ Administration Bureau of Dongzhai National Nature Reserve Luoshan Henan Province China; ^4^ Key Laboratory of Animal Ecology and Conservation Biology, Institute of Zoology Chinese Academy of Sciences Chaoyang Beijing China; ^5^ Cardiff University ‐ Institute of Zoology Joint Laboratory for Biocomplexity Research Chinese Academy of Sciences Beijing China

**Keywords:** candling, conservation management, crested ibis ( *Nipponia nippon* ), embryonic development, endangered species

## Abstract

Identifying key factors influencing the survival of animals, particularly rare and endangered species, is crucial to biodiversity conservation. In birds, hatching failure is pronounced in endangered species. Accurate assessment of egg development and the ability to distinguish nonviable eggs are essential prerequisites for evaluating the impact of factors that lead to hatching failure and applying appropriate conservation practices. The crested ibis ( *Nipponia nippon* ), a flagship endangered species, has a long history of captive breeding, which has contributed to population recovery. However, little is known about the specific process and characteristics of its egg development. In this study, we provided the first comprehensive description of normal egg development in the crested ibis, including both the changes observed in unfertilized eggs during incubation and embryonic development via candling (*n* = 98 eggs; total = 1422 candling images; mean ± SE = 14.52 ± 1.38 images per egg), and offered a practical reference for assessing fertilization status and embryo viability. In addition, we estimated the timing of embryo mortality and found that most deaths occurred during mid‐incubation (Day 7–15) (*n* = 12, 60.0%) and shortly before hatching (Day 23–29) (*n* = 7, 35.0%), highlighting these critical periods that require particular attention. This species‐specific documentation of egg development provides a valuable reference for accurately assessing embryonic progress, evaluating environmental effects on survival, and guiding adaptive management, benefiting both captive breeding programs and field conservation efforts.

## Graphical abstract

Using candling images from 98 eggs, we present the first detailed description of egg and embryonic development in the endangered crested ibis. We identify diagnostic features of nonviable eggs and reveal that embryo mortality is concentrated during mid‐incubation and shortly before hatching. These findings provide a standardized reference for assessing embryonic viability and investigating the ecological and evolutionary drivers of reproductive failure in threatened bird populations.

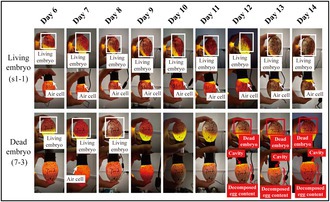

## 1 Introduction

Biodiversity loss has become an urgent global concern, calling for effective conservation actions, particularly for rare and endangered species. Birds represent the most diverse group of vertebrates, yet approximately 11% of avian species assessed by the IUCN are currently threatened with extinction (IUCN 2025). Identifying the key factors influencing their survival is therefore of paramount importance for developing effective conservation strategies. Egg development is crucial for survival in birds. Both in field studies and captive breeding programs, researchers have frequently observed that hatching failure is common, especially among endangered species that have experienced a genetic bottleneck and inbreeding (Koenig 1982; Briskie and Mackintosh 2004; Marshall et al. 2023). Hatching failure generally results from either infertility or embryonic death, which can be attributed to intrinsic factors such as the quality of parental gametes (Donoghue 1999; Assersohn et al. 2021), nutritional imbalances in parents (Wilson 1997), and congenital diseases caused by inbreeding (Fu et al. 2019), or extrinsic influences including inappropriate temperature and humidity, bacterial infection, and mechanical disturbance during handling or movements of eggs (Ori 2011; Rideout 2012). It is essential to distinguish viable from nonviable eggs and further assess normal embryonic development. In doing so, we can determine the age of eggs, predict hatch date, identify whether hatching failure stems from fertility or embryo mortality, remove abnormal eggs before contamination occurs, pinpoint critical periods during incubation when embryo mortality is concentrated, so to determine specific impacts on development, and guide appropriate actions to improve breeding success. A comprehensive understanding of egg development is thus one of the prerequisites for diagnosing the causes of hatching failure and improving conservation outcomes for endangered species.

As the most diverse vertebrate group, birds exhibit substantial interspecific variation in egg size, incubation duration, hatchling mass, and developmental maturity (altricial vs. precocial) (Starck and Ricklefs 1998; C. Deeming 2007; Cooney et al. 2020). Correspondingly, species exhibit great variation in embryonic development as well (Daniel Jr. 1957; Blom and Lilja 2005). Species‐specific descriptions of embryonic development are essential to guide further research and improve species management. To date, the complete or near‐complete egg (embryonic) developmental process has been documented in many avian species using candling, breakout examination, and molecular techniques. For example, detailed anatomical studies have been conducted in species of commercial or research importance, such as chicken (*Gallus gallus domesticus*) (Hamburger and Hamilton 1951), pigeon ( *Columba livia* ) (Łukasiewicz 2014), Japanese quail (*Coturnix coturnix japonica*) (Ainsworth et al. 2010), Guinea fowl ( *Numida meleagris* ) (de Araújo et al. 2019), Peking duck (*Anas platyrhynchos domestica*) (Trela et al. 2025), and turkey ( *Meleagris gallopavo* ) (Mun and Kosin 1960). Also, studies have been performed on representative non‐domestic taxa with significant evolutionary or ecological importance, such as emu ( *Dromaius novaehollandiae* ) (Nagai et al. 2011) and ostrich ( *Struthio camelus* ) (Deeming 1995), whose embryonic traits can provide insights into the evolution and conservation of developmental characteristics across birds. In addition, species such as American kestrel ( *Falco sparverius* ), a common North American raptor which is frequently used in experimental studies as a model raptor species (Bird et al. 1984; Pisenti et al. 2024), and zebra finch ( *Taeniopygia guttata* ), another famous model passerine species that has been widely used in avian research, have been carefully studied (Hemmings and Birkhead 2016; Pei et al. 2020), along with other taxa including blue tit ( *Cyanistes caeruleus* ) (Hemmings and Birkhead 2016), great tit ( *Parus major* ) (Hemmings and Birkhead 2016), pheasant ( *Phasianus colchicus* ) (Fant 1957), society finch (*Lonchura striata domestica*) (Yamasaki and Tonosaki 1988), mallard ( *Anas platyrhynchos* ) (Caldwell and Snart 1974), Adélie penguin ( *Pygoscelis adeliae* ) (Herbert 1967), and bobwhite quail ( *Colinus virginianus* ) (Hendrickx and Hanzlik 1965). Nevertheless, most documented species belong to species with commercial importance or laboratory model species. Taxonomically, they belong to Passeriformes, Galliformes, or Anseriformes. In contrast, little is known about egg or embryonic development in endangered species or other taxa, especially flagship species that experienced a serious bottleneck. In fact, investigations on embryonic development in these endangered species are of both evolutionary and conservation importance, as they may help reveal the causes of stage‐specific embryonic mortality, link developmental patterns with existing genetic evidence, and inform improvements in conservation and management practices.

Crested ibis ( *Nipponia nippon* ) is an iconic and flagship endangered species (Pelecaniformes) that has recovered from an extremely small population (Liu 1981; Li 2023). This species was historically widespread across Northeast Asia (BirdLife‐International 2001; Li et al. 2014), while in the 20th century, its population declined significantly as a result of habitat destruction and human activity (e.g., illegal hunting, overuse of fertilizer and pesticide) (Archibald et al. 1980; Ding 2004; Li et al. 2009; Feng et al. 2019). It was once presumed extinct after populations in Russia, Korea and Japan consecutively died off (Archibald and Lantis 1979; Anderson 1984). In 1981, a small wild population of seven individuals, including two breeding pairs and three chicks belonging to one of the couples, was rediscovered in Yangxian County of Shaanxi Province, China (Liu 1981). Since then, extensive conservation efforts have been implemented to save this endangered species, and so far, the global population of wild and captive crested ibis is estimated to exceed 10,000 (Li 2023). Nonetheless, as a species recovered from an extremely small population, it still suffers from inbreeding depression and low genetic diversity owing to the limited number of founders, which are already believed to contribute to reduced embryo survival and individual fitness (Fu et al. 2019; Zheng et al. 2026). To better understand the effects of factors such as inbreeding on crested ibis and to inform appropriate conservation measures, it is essential to accurately identify the fertilization status of eggs and the developmental stage at which embryo mortality occurs. Despite remarkable progress in population recovery, including long‐term successful captive breeding, detailed knowledge of egg development in this species remains limited. Earlier studies on crested ibis reproduction mainly focused on population growth and husbandry improvements, providing quantitative data such as clutch size, fertilization rate, and hatching rate (Xi et al. 2001; Huang et al. 2016) or practical information on incubation and chick care (Liu et al. 1999; Ding 2004; Huang et al. 2006). Aside from one report that summarized embryonic mortality rates across developmental stages and included a few photographs of dead embryos (Yang et al. 2020), investigation details of egg and embryonic development have been hardly described in literature.

Candling is a long‐established, noninvasive method for assessing egg fertility and embryonic status in both captive and wild populations (Westerskov 1950; Weller 1956; Mauldin 2002; Ernst et al. 2004). It involves using a flashlight or specialized device with a cool light source held against the blunt end of the egg (Hall et al. 2023). By examining blood vessel patterns, the silhouette of the embryo, the size of the air cell, and changes in opacity, researchers can monitor embryonic development. Unfertilized eggs or dead embryos exhibit distinct visual features, enabling early identification and removal of nonviable eggs (Deeming 1995; Mauldin 2002; Ernst et al. 2004). Proper candling has been shown to have minimal effect on hatching success (Reis and Soares 1993). Negative impacts generally result from poor handling practices, such as excessive exposure to room temperature, the use of nonspecialized heated light sources, or mechanical shock. When performed correctly, candling is a reliable and safe diagnostic tool during incubation. Although its effectiveness decreases in species with thick‐shelled or darkly pigmented eggs (Westerskov 1950), as a noninvasive and convenient identification method, candling remains highly valuable for conservation programs, especially in rare species such as the crested ibis that require strict protection.

In this study, we provided the first comprehensive description and assessment of egg development, which included both the changes observed in unfertilized eggs and embryonic development in the crested ibis based on the candling images collected throughout the entire incubation period. By analyzing extensive images of eggs with different fates, we aimed to (1) describe and illustrate the details of development stages in normal eggs; (2) identify key diagnostic features that distinguish nonviable eggs, including infertile eggs and dead embryos, from viable eggs; and (3) estimate the death times of embryos to identify critical periods that require greater attention in breeding programs, based on the information of embryos with accurately recorded death times and characteristic differences observed in candling images. Species‐specific documentation of egg development is necessary not only for determining whether hatching failure results from infertility or embryonic death, but also for estimating the timing of embryo loss, evaluating environmental effects on survival, and guiding adaptive management. For endangered species such as the crested ibis, which had experienced a severe population bottleneck, comprehensive knowledge and accurate measurement of fertilization and embryo mortality are the prerequisites for diagnosing the causes of hatching failure, quantifying inbreeding depression, and ultimately improving reproductive outcomes. Such information is valuable for both captive breeding and field conservation efforts. Overall, our findings provide an important addition to existing studies of avian embryonic development. Also, it may help guide future research while offering insights into the evolution and conservation of developmental traits across birds.

## 2 Materials and Methods

### 2.1 Study Species

**FIGURE 1:**
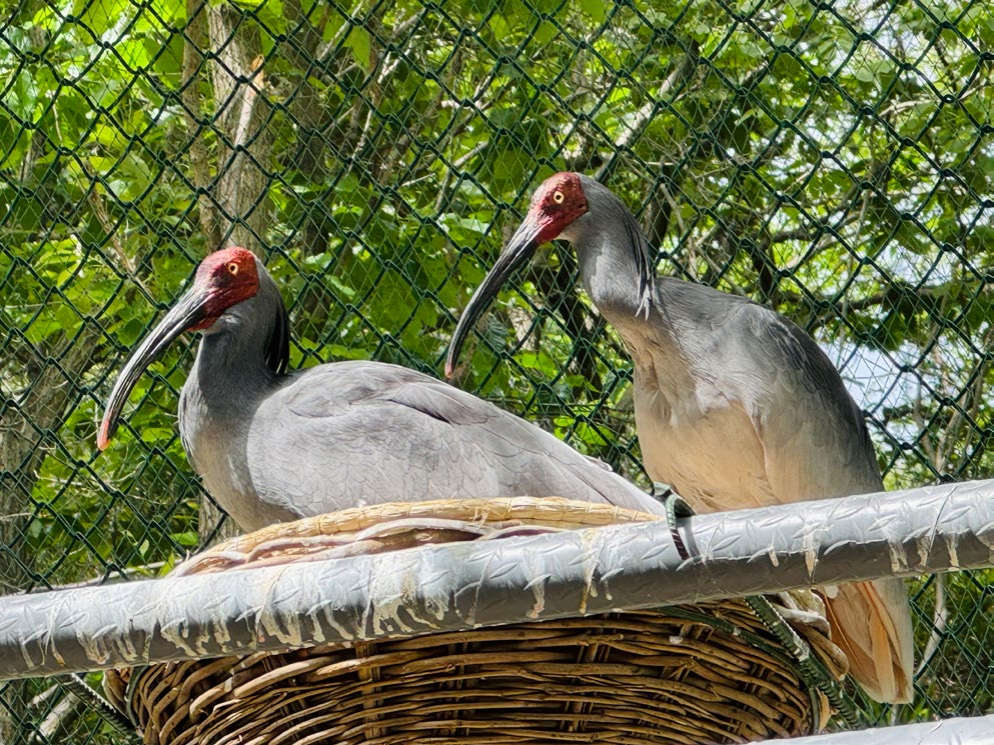
A pair of crested ibis during the breeding season, with the male on the left and the female on the right. Photo by Yuansi He.

Crested ibis is a medium‐sized wading bird characterized by red facial skin and legs, and predominantly white plumage with orange‐cinnamon tones on the tail, abdomen and flight feathers. During the breeding season, they secrete a black substance from a patch of skin in the neck and throat region, and apply this substance to their crest, scapulars, mantle and wings through ‘daubing behavior’ (Figure 1). Individuals typically reach sexual maturity at around 3 years old (ranging from 2 to 4 years) (Yu et al. 2010). The breeding season generally extends from late February to late June, with mating and nest‐building occurring from late February to early March, followed by egg‐laying from mid‐March to early April (Li and Huang 1986; Shi et al. 1989; Wang and Shi 1999). The incubation period lasts about 28 days, and it usually takes 30–40 h for the chick to fully hatch out after breaking the egg (Shi et al. 1999; Zhai et al. 1999; Huang et al. 2016). A breeding pair usually lays one clutch per year, but may produce a replacement clutch if the first is lost early in the season. Each clutch contains 2–4 eggs, laid at 1‐ or 2‐day intervals (Shi et al. 1999). While in captivity, egg production can exceed natural levels—sometimes reaching more than 10—due to egg collection, which stimulates additional laying. The crested ibis is altricial. At the time of hatching, its eyes have not yet opened, with sparse down feathers covering its body, and its legs are weak. Chicks need to be raised by adult birds until they are 40–45 days old before they can leave the nest (Shi et al. 1999; Zhai et al. 1999).

### 2.2 Egg Collection and Incubation

Eggs were collected from the captive crested ibis population during the 2025 breeding season at the Crested Ibis Breeding Center at Dongzhai National Nature Reserve, Henan Province, China. All eggs (*n* = 98) were initially incubated naturally by their parents. The average duration of natural incubation before the eggs were transferred to the artificial incubator was 4 ± 0.54 days (mean ± SE, range 0 to 23). Among these, 78 eggs (80%) were moved to artificial incubation between Day 0 and Day 5 of incubation, 8 eggs (8%) between Day 6 and Day 11, 8 eggs (8%) between Day 12 and Day 17, and 4 eggs (4%) between Day 18 and Day 23. Prior to incubation, each egg was disinfected by immersion in warm water containing potassium permanganate and gently dried. Artificial incubation was carried out in hatching incubators (P‐008A‐TOKI52, Japan; Rcom Pro 20, Korea) at 37.5°C–37.7°C and 55%–65% relative humidity. To simulate natural cooling behavior, eggs were removed from the incubator every 3 h between 8:00 and 19:00, cooled for 3 min, and then returned.

### 2.3 Egg Candling

Eggs were candled daily using a dedicated egg candler (Zhenye (Huizhou) Industrial Co. Ltd., Huizhou, China), which does not generate heat during the examination, thereby minimizing potential harm to the embryos. Candling was conducted in darkness from both the sharp and blunt ends of each egg and completed in less than 3 min. Eggs were promptly returned to the incubators after images were taken using an iPhone 15 Pro (Apple Inc., USA). After Day 25 of incubation, when hatching was imminent, candling was discontinued for most eggs to avoid disturbing the hatching process, except for a few infertile eggs and those showing no signs of pipping. Throughout the whole incubation period, we carried out the following practices: (1) eggs that emitted a foul odor due to decomposition or showed no signs of embryonic development after 7 days were opened to assess fertility; (2) unhatched eggs remaining after the expected hatching date, or eggs in which the embryo appeared nonviable, were dissected to determine developmental status; (3) for embryos that died during incubation without a precisely known time of death, the approximate timing was inferred from embryo size at necropsy combined with candling records obtained during the incubation period.

### 2.4 Picture Processing

**FIGURE 2:**
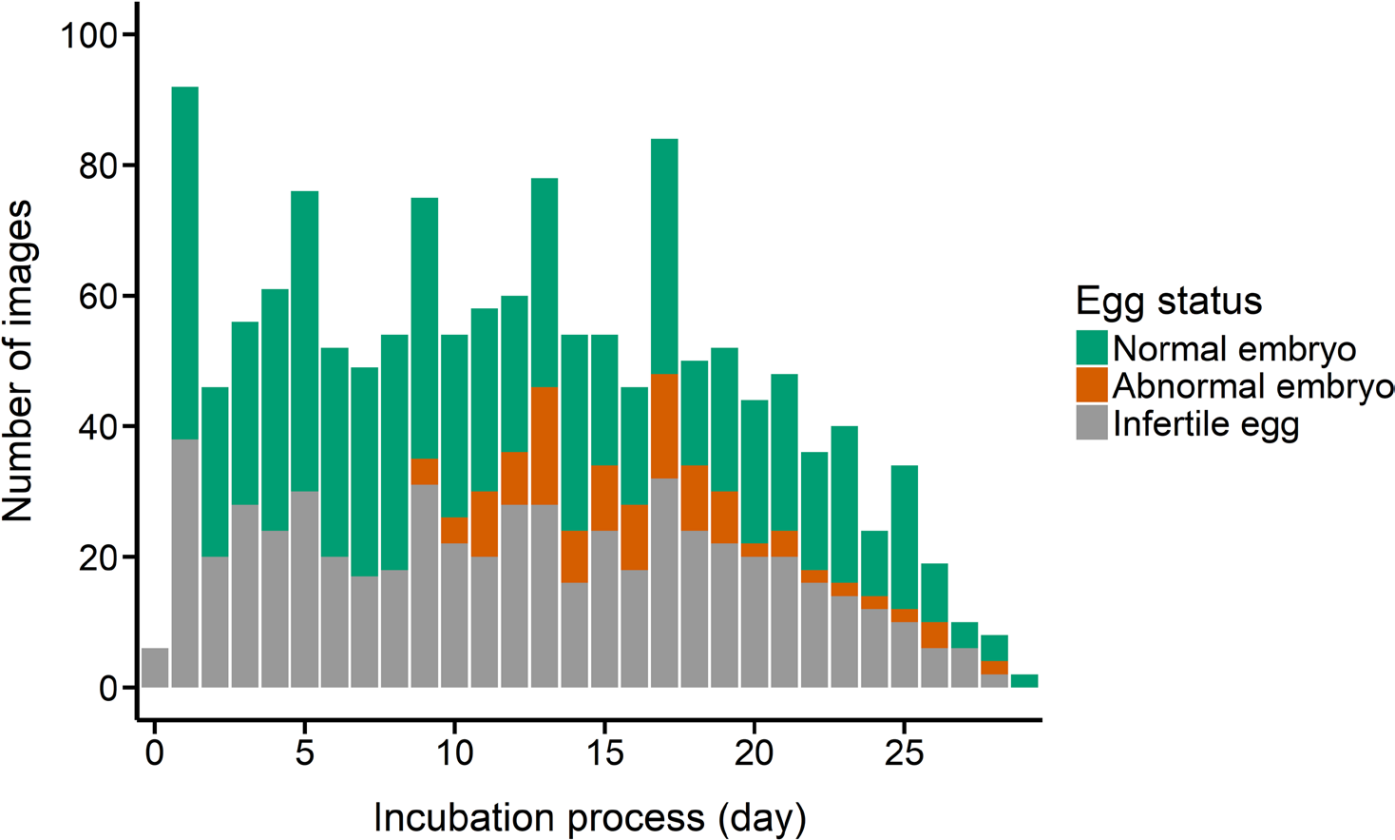
Number of images taken throughout the incubation. Day 0 referred to the day when the egg was laid. For eggs in which the embryos eventually died, images taken before any visible signs of abnormality appeared were classified as normal embryos.

A total of 1422 images of 98 eggs were taken during the breeding season (Table 1). Images were taken throughout the entire incubation process, ensuring the comprehensive monitoring of egg development (Figure 2, Table S1). All images were cropped and arranged using Adobe Photoshop (Adobe Inc., USA) and Adobe Illustrator (Adobe Inc., USA). Since no scale bars were included during photography, image scales were calibrated using objects of known size present in the images (e.g., operator's hands, sample tubes).

**TABLE 1:** Summary of egg classification, total number of images and average number of images during incubation.

Destiny		Number of eggs (%)	Total number of images	Average number of images (±SE)
Infertile		46 (47%)	572	12.43 ± 2.18
Abnormal developing embryo		19 (19%)	368	19.37 ± 3.10
Normally developing embryo	Accidentally died	3 (3%)	16	5.33 ± 1.76
	Abnormal positioning	3 (3%)	105	35 ± 12.12
	Hatched	27 (28%)	361	13.37 ± 1.63
Total		98	1422	14.52 ± 1.38

The images were first classified into different categories based on the final incubation outcomes and the developmental conditions (i.e., infertile, dead embryo, successfully hatched) determined by breakout examination, which referred to breaking out the egg from the blunt end and examining whether there was an embryo in it or signs like blood that indicated embryo development. Eggs were classified as *infertile* if no embryonic structures or blood vessels were observed upon breakout examination, as *dead embryo* if embryonic tissue or vascular structures were present without successful hatching, and as *successfully hatched* if chicks emerged. To describe and illustrate the developmental details at different stages in normal eggs, we compiled all the candling images of eggs that developed normally (including hatched eggs and those that failed due to accidental breakage or abnormal positioning) to summarize their changing characteristics throughout incubation, and selected representative images for annotation and explanation. Subsequently, to address the key question of how to identify nonviable eggs, including infertile eggs and dead embryos, we compared candling images of normally developing eggs and abnormal eggs (unfertilized or containing dead embryos) at corresponding stages, summarized the key distinguishing features, and chose representative images to illustrate and describe these characteristics. Finally, to determine the timing of embryo death, we first estimated the approximate time of death based on the appearance of abnormalities in the candling images. A more precise estimation was then obtained by comparing the morphological characteristics of the dead embryos after breakout examination with those of four embryos whose death times were clearly recorded due to accidental events. The distribution of death events was subsequently analyzed to identify the critical periods of embryonic development in the crested ibis. Graphical presentations of the results were produced using the ggplot2 (Wickham 2016) package in R 4.5.1 (R Core Team 2022).

### 2.5 Validation of Diagnostic Features

To assess the diagnostic objectivity and strength of these candling features, we conducted validation analyses for two key applications: classification of developmental stage and the identification of nonviable eggs. It should be noted that subsequent references to features, such as “shadow” and “vascular structures,” refer to descriptive visual patterns observed during candling and are not intended to represent anatomically verified structures, as definitive anatomical identification cannot be made from candling observations alone.

#### 2.5.1 Classification of Developmental Stage

To evaluate whether the candling features can help distinguish developmental stages reliably (i.e., six developmental stages based on stage‐specific features as described in the Results: (1) Day 1–3, (2) Day 4–6, (3) Day 7–12, (4) Day 13–16, (5) Day 17–20, and (6) Day 21–24), we asked three observers (all with undergraduate degrees, including one with a background in biology) to independently classify the eggs based on the diagnostic candling features and reference images provided in the Results (*Development stages in normal eggs*). Then we assessed both their classification accuracy and interobserver agreement to evaluate the effectiveness of the features.

A total of 483 candling images of normally developing embryos were initially collected. As described above, each egg was candled and photographed from both the blunt and sharp ends. Eggs lacking paired views and those used as reference examples were excluded. And because chicks begin pipping after Day 25, which results in fewer records and allows hatching progress to be inferred directly, only eggs from Day 1 to Day 24 of incubation were retained. After filtering, 342 images (171 eggs) remained. All images were anonymized by concealing candling dates and incubation days, and 57 eggs were randomly selected as the test dataset. During the classification, observers were provided with paired images of each egg, along with descriptions and reference images of stage‐specific features (as described in the Section 3), and were asked to assign each egg to one of the six developmental stages.

Classification performance was evaluated using accuracy (i.e., proportion of exact agreement between observer classifications and the true stage) and mean absolute error (i.e., average absolute deviation from the true stage). Given the ordinal nature of the stages, Krippendorff's α and weighted Cohen's kappa were calculated using the irr package (Gamer et al. 2019) to assess the agreement between observer classifications and the true stages, as well as interobserver agreement.

#### 2.5.2 Identification of Nonviable Eggs

To assess the diagnostic and predictive performance of the proposed candling features in relation to observed egg outcomes, we asked three additional observers (all with undergraduate degrees in non‐biological disciplines, including one currently pursuing a graduate degree in ecology) to assess the eggs based on the diagnostic candling features and reference images presented in the Results (*Identifying nonviable eggs*). They were asked to determine fertilization status (1 = fertilized, 0 = unfertilized), and, if fertilized, embryo viability (1 = alive, 0 = dead; NA for unfertilized eggs), and to record the presence (1) or absence (0) of the diagnostic features provided. We then assessed observer classification accuracy and interobserver agreement to evaluate feature performance, and used generalized linear mixed models to examine the association between diagnostic features and true fertilization status as well as embryo viability.

Since no clear differences between fertilized and unfertilized eggs were detectable during Day 1 to Day 3 of incubation, only eggs from Day 4 onward were assessed. Eggs from Day 4–6 were assessed for fertilization status only. In this assessment, 191 candling images were available. After excluding eggs lacking paired views and those used as reference examples, 158 images (79 eggs) remained, of which 40 eggs were randomly selected as the test dataset. On the other hand, eggs from Day 6 to 24 were evaluated for both fertilization status and, if fertilized, embryo viability. In this dataset, 1060 candling images were initially available, and 758 images (379 eggs) remained after applying the same exclusion criteria. From these, 190 eggs were randomly selected as the test dataset.

For Day 4–6, diagnostic features included the presence of a broad shadow and distinct shadow edge when candled from the sharp and blunt ends, denoted as *s_broad_shadow*, *s_shadow_with_distinct_edge*, *b_broad_shadow*, and *b_shadow_with_distinct_edge*, respectively. For Day 6–24, apart from those mentioned above, additional features included shadow coverage exceeding 50% (*s_shadow_coverage_gt50*), a uniform dark shadow (*s_uniform_dark_shadow*), and vascular structures (*s_vascular_structure*) when candled from the sharp end, as well as vascular structures (*b_vascular_structure*) and an air cell with distinct edge (*b_aircell_with_distinct_edge*) when candled from the blunt end.

Classification accuracy (i.e., proportion of exact agreement between observer classifications and the true status) and diagnostic performance (i.e., true positives, true negatives, false positives, and false negatives) were calculated for evaluating observer accuracy in classifying fertilization and embryo viability. Interobserver agreement was assessed using the irr package (Gamer et al. 2019). Additionally, because misclassification of fertilization status propagates errors in viability assessment, cases in which unfertilized eggs were incorrectly classified as fertilized were excluded from analyses of embryo viability. To evaluate the diagnostic value of each feature, generalized linear mixed models were fitted using the lme4 package (Bates et al. 2015), with true fertilization status or embryo viability as the binary response variable, diagnostic features as fixed effects, observer identity as a random effect, and a binomial error distribution. For embryo viability models (Day 6–24), features with near‐zero variance, defined as having more than 95% of observations in a single category, were excluded to avoid model instability. This led to the exclusion of broad shadow features (*s_broad_shadow* and *b_broad_shadow*).

All of the analyses were performed in R 4.5.1 (R Core Team 2022).

## 3 Results

### 3.1 General Summary

A total of 98 eggs were collected and candled during the breeding season, of which 52 (49%) were fertilized and 27 successfully hatched (25%). Among the fertilized eggs that failed to hatch (*n* = 25), three were accidentally broken during handling, and three failed to pip the eggshell because of abnormal fetal positioning, resulting in death from asphyxiation. In total, 1422 images were taken during the incubation period, including 482 images documenting the developmental process of normally developing embryos (including embryos that later failed due to accidents or abnormal positioning), 368 documenting embryos that died during development, and 572 documenting the incubation process of infertile eggs (Table 1). In addition, 24 images of dead embryos were taken after egg opening (including one naturally incubated egg that broke because the nest collapsed), allowing partial insight into embryonic morphology at different developmental stages in the crested ibis.

### 3.2 Development Stages in Normal Eggs

The text below describes the candling images of normally developing embryos during the incubation process, which can serve as references for assessing fertilization status, estimating embryonic age, and evaluating embryonic viability. For each egg, the images on the top were taken when candling from the sharp end, and those on the bottom were taken when candling from the blunt end. In the following descriptions of candling observations, features such as “shadow” and “vascular structures” are noted as visual patterns detectable in the images. These terms are used descriptively and do not imply confirmed anatomical structures, as candling alone cannot provide definitive anatomical identification.

#### 3.2.1 Day 1–3

**FIGURE 3:**
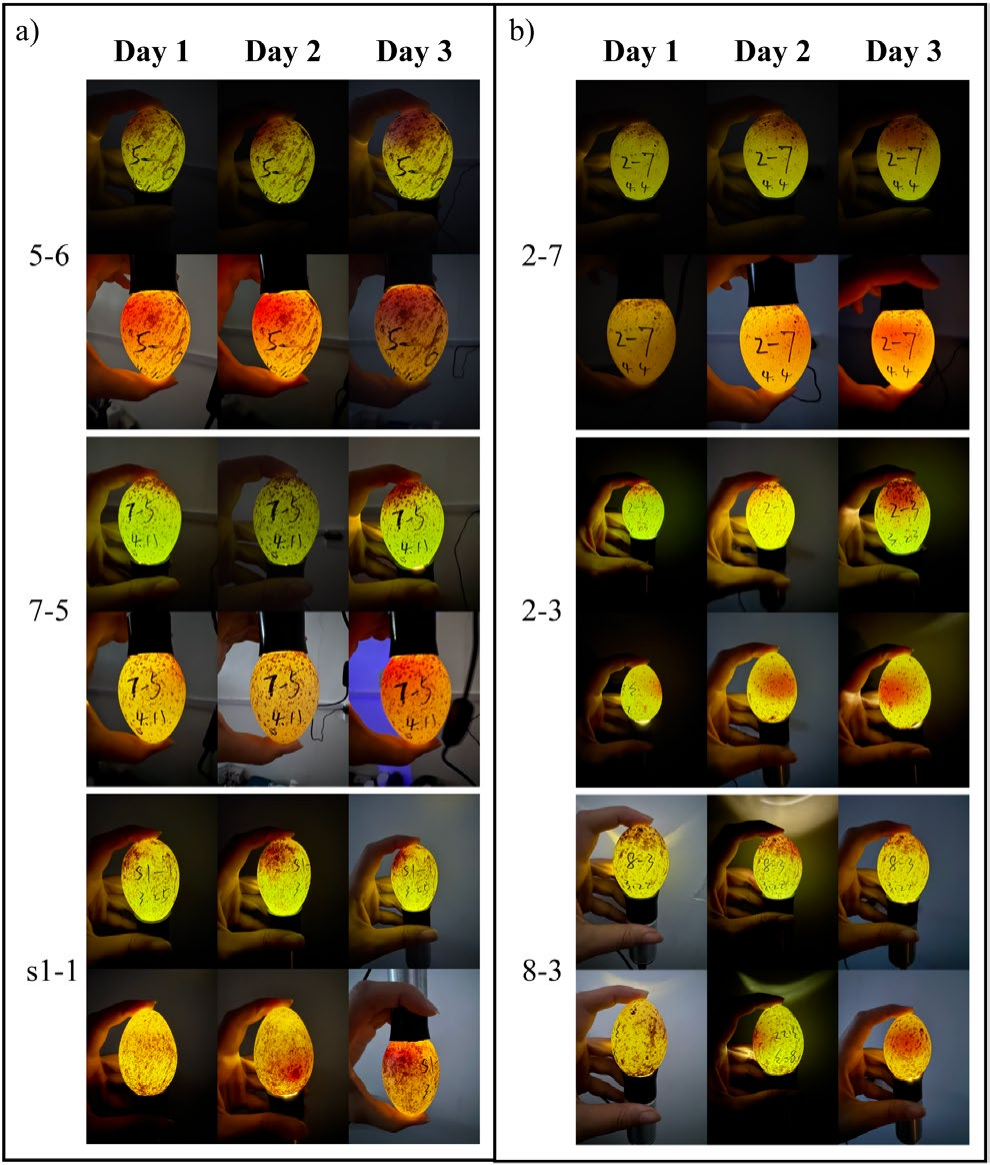
Representative candling images of fertile (a) and infertile (b) eggs during Day 1 to Day 3 of development. For each egg (5‐6, 7‐5, s1‐1, 2‐7, 2‐3, 8‐3), the images on the top were taken when candling from the sharp end, and those on the bottom were taken when candling from the blunt end.

Fertilized (*n* = 31 eggs, 108 images; Figure 3a) and unfertilized (*n* = 26 eggs, 86 images; Figure 3b) eggs appear indistinguishable regardless of the candling end, with complete transparency and only a vague shadow without clear boundaries.

#### 3.2.2 Day 4–6

**FIGURE 4:**
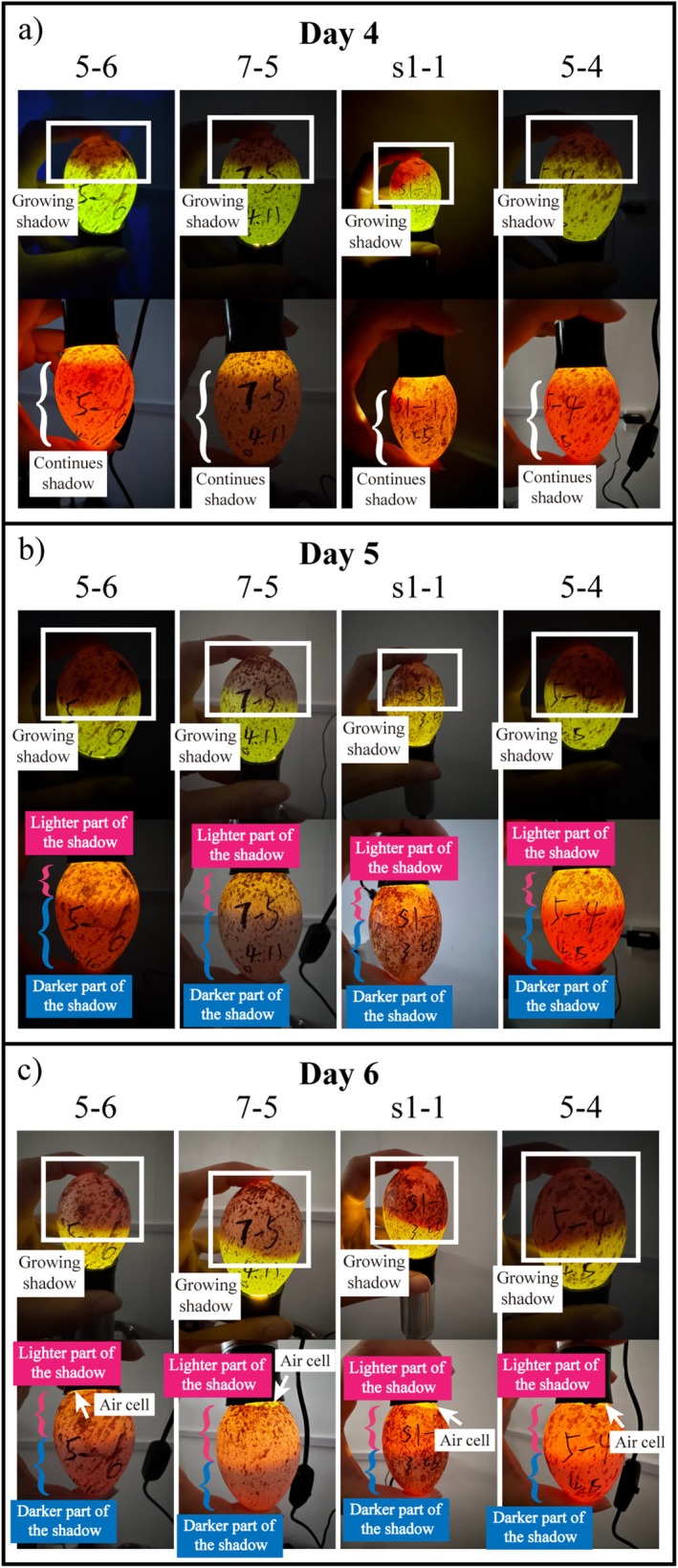
Representative candling images of fertile eggs during Day 4 and Day 6 of development. The growing shadows at the blunt end were highlighted in the white boxes. The white braces showed the continuous shadows at the sharp end. The darker and lighter parts of the shadow were pointed out by blue and pink braces separately. The white arrows showed the air cell at the blunt end. For each egg (5‐6, 7‐5, s1‐1, 5‐4), the images on the top were taken when candling from the sharp end, and those on the bottom were taken when candling from the blunt end.

A distinct shadow becomes visible at the blunt end when candled from the sharp end (Figure 4a–c, white boxes), indicating the onset of embryonic development. From the blunt end, the shadow at the sharp end appears as a continuous area rather than a floating sphere (Figure 4a, white braces). This shadow is typically darker at the bottom (Figure 4b,c, dark blue braces), lighter at the top (Figure 4b,c, pink braces), and a transparent region corresponding to the air cell is visible at the blunt end (Figure 4c, white arrows).

#### 3.2.3 Day 7–12

**FIGURE 5:**
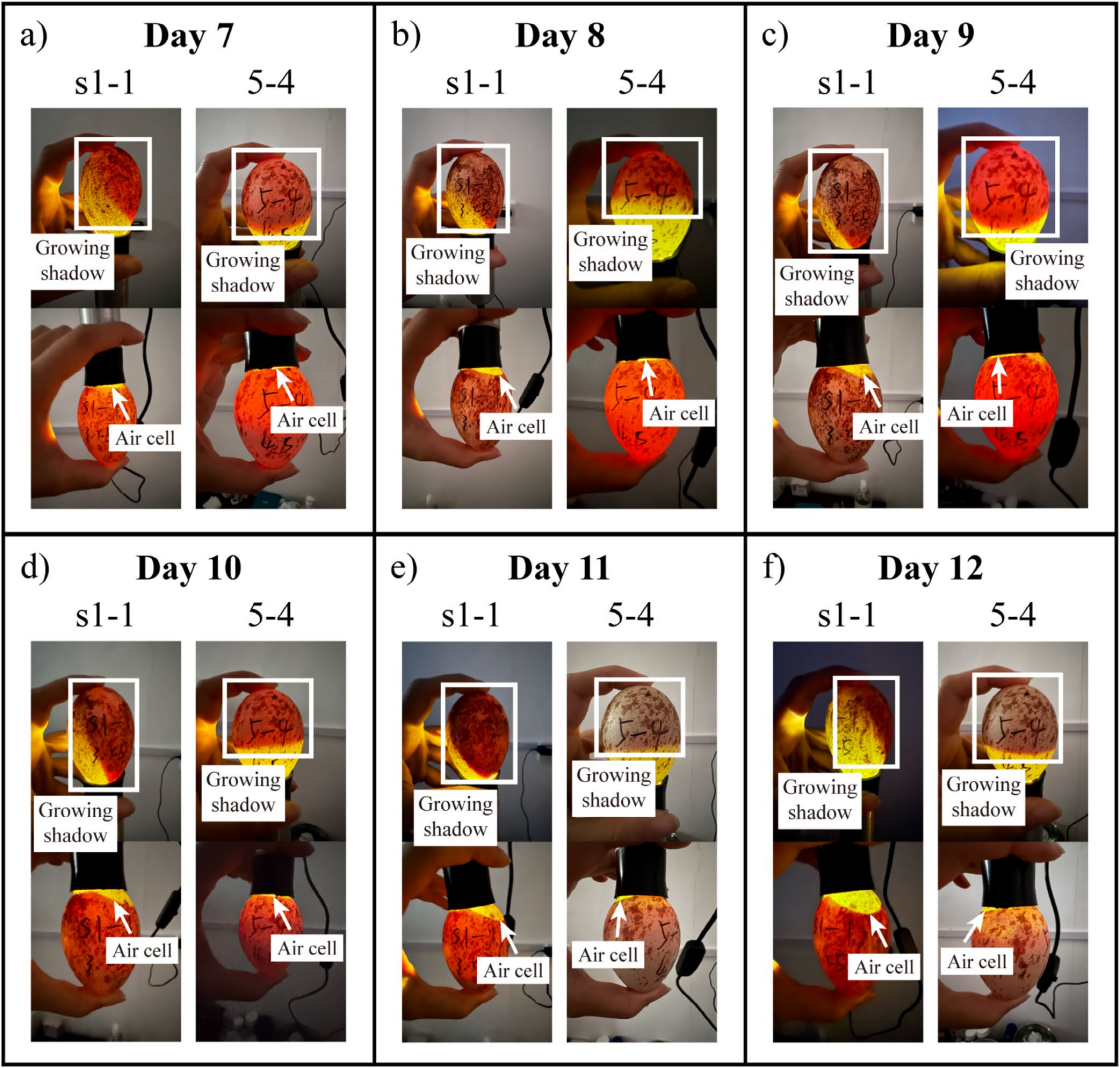
Representative candling images of fertile eggs during Day 7 and Day 12 of development. The shadows at the blunt end were highlighted in the white boxes. The air cells were pointed out by white arrows. For each egg (s1‐1, 5‐4), the images on the top were taken when candling from the sharp end, and those on the bottom were taken when candling from the blunt end.

The shadow at the blunt end when candled from the sharp end expands rapidly, occupying approximately half of the egg (Figure 5a–f, white boxes). The position of the embryos may change if the incubation posture changes (e.g., from vertical incubation with the blunt end upward to horizontal incubation in a flat position). Meanwhile, the air cell gradually enlarges and develops a distinct and well‐defined boundary (Figure 5a–f, white arrows).

#### 3.2.4 Day 13–16

**FIGURE 6:**
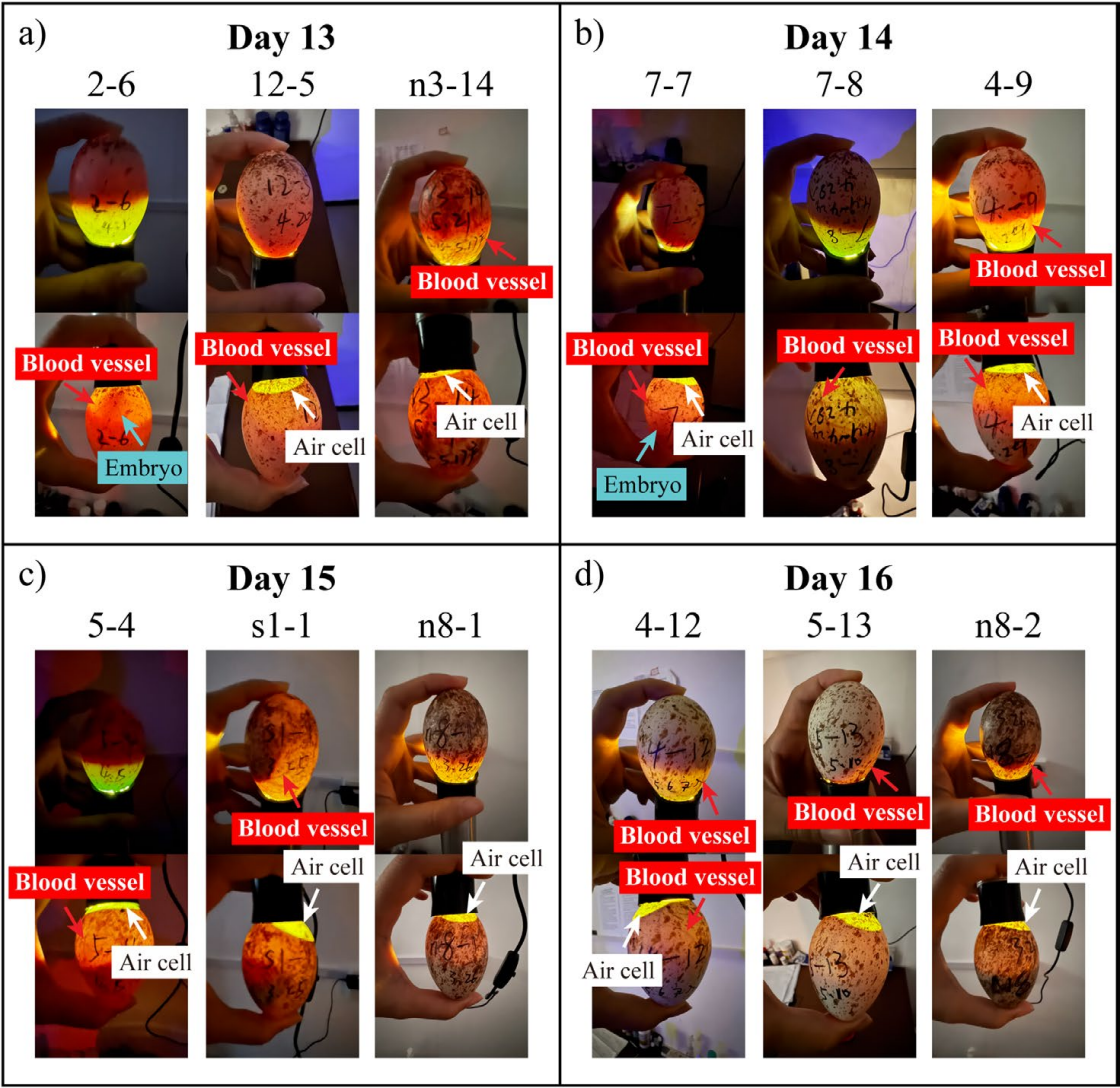
Representative candling images of fertile eggs during Day 13 and Day 16 of development. The red arrows showed vascular structures around the shadows. The white arrows pointed out the air cells. The embryonic movements were pointed out by light blue arrows. For each egg (2‐6, 12‐5, n3‐14, 7‐7, 7‐8, 4‐9, 5‐4, s1‐1, n8‐1, 4‐12, 5‐13, n8‐2), the images on the top were taken when candling from the sharp end, and those on the bottom were taken when candling from the blunt end.

Vascular structures become visible along the margins of the shadow when candled from either end of the egg (Figure 6a–d, red arrows). The air cell remains stable and clearly visible (Figure 6a–d, white arrows). When candled from the sharp end, occasional embryonic movements are observable (Figure 6a,b, light blue arrows).

#### 3.2.5 Day 17–20

**FIGURE 7:**
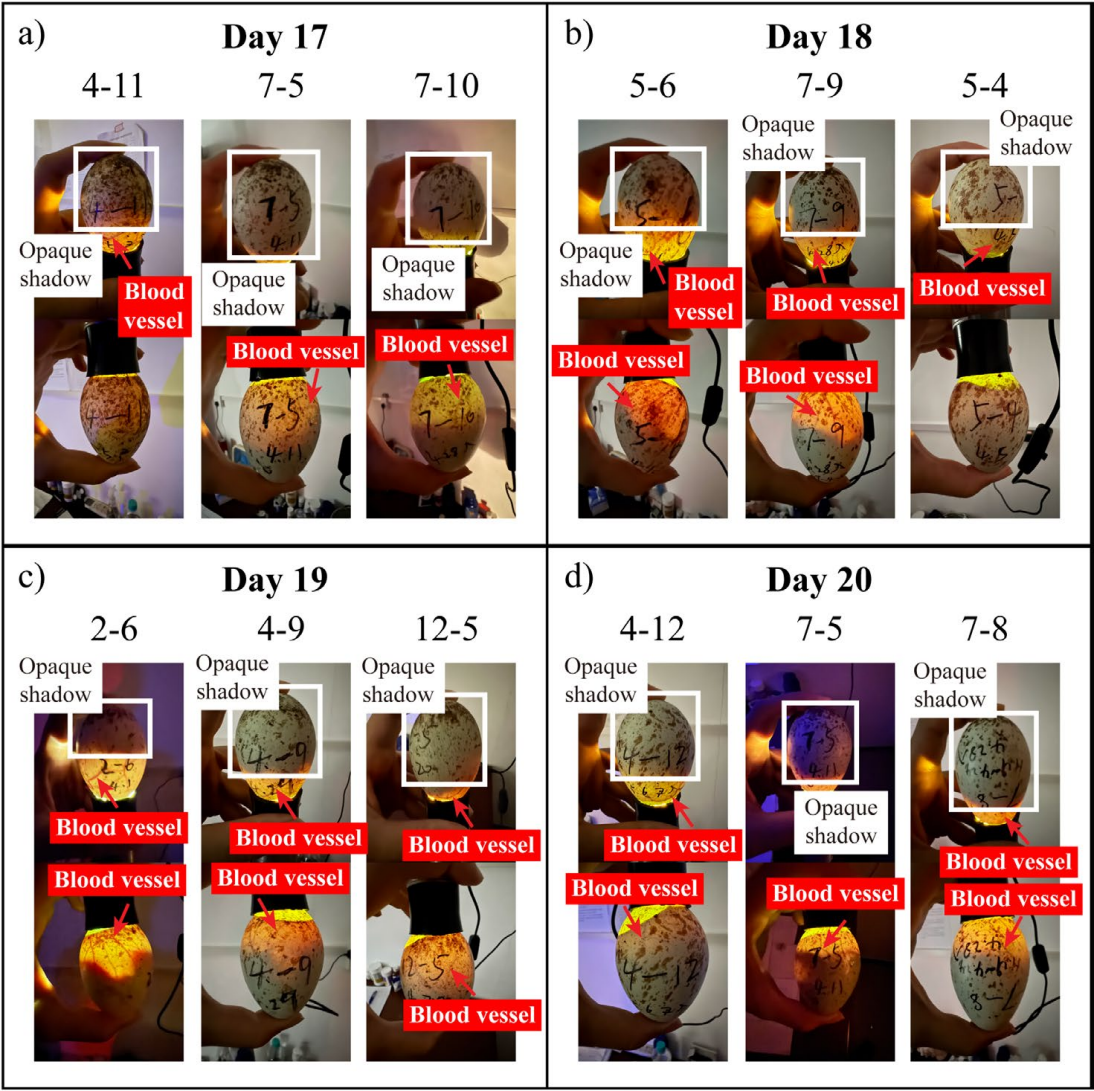
Representative candling images of fertile eggs during Day 17 and Day 20 of development. The white boxes showed the shadows at the blunt end. The red arrows pointed out the vascular structures. For each egg (4‐11, 7‐5, 7‐10, 5‐6, 7‐9, 5‐4, 2‐6, 4‐9, 12‐5, 4‐12, 7‐8), the images on the top were taken when candling from the sharp end, and those on the bottom were taken when candling from the blunt end.

The shadow at the blunt end when candled from the sharp end becomes opaque, losing its reddish translucency (Figure 7a–d, white boxes), and vascular structures become more prominent (Figure 7a–d, red arrows).

#### 3.2.6 Day 21–24

**FIGURE 8:**
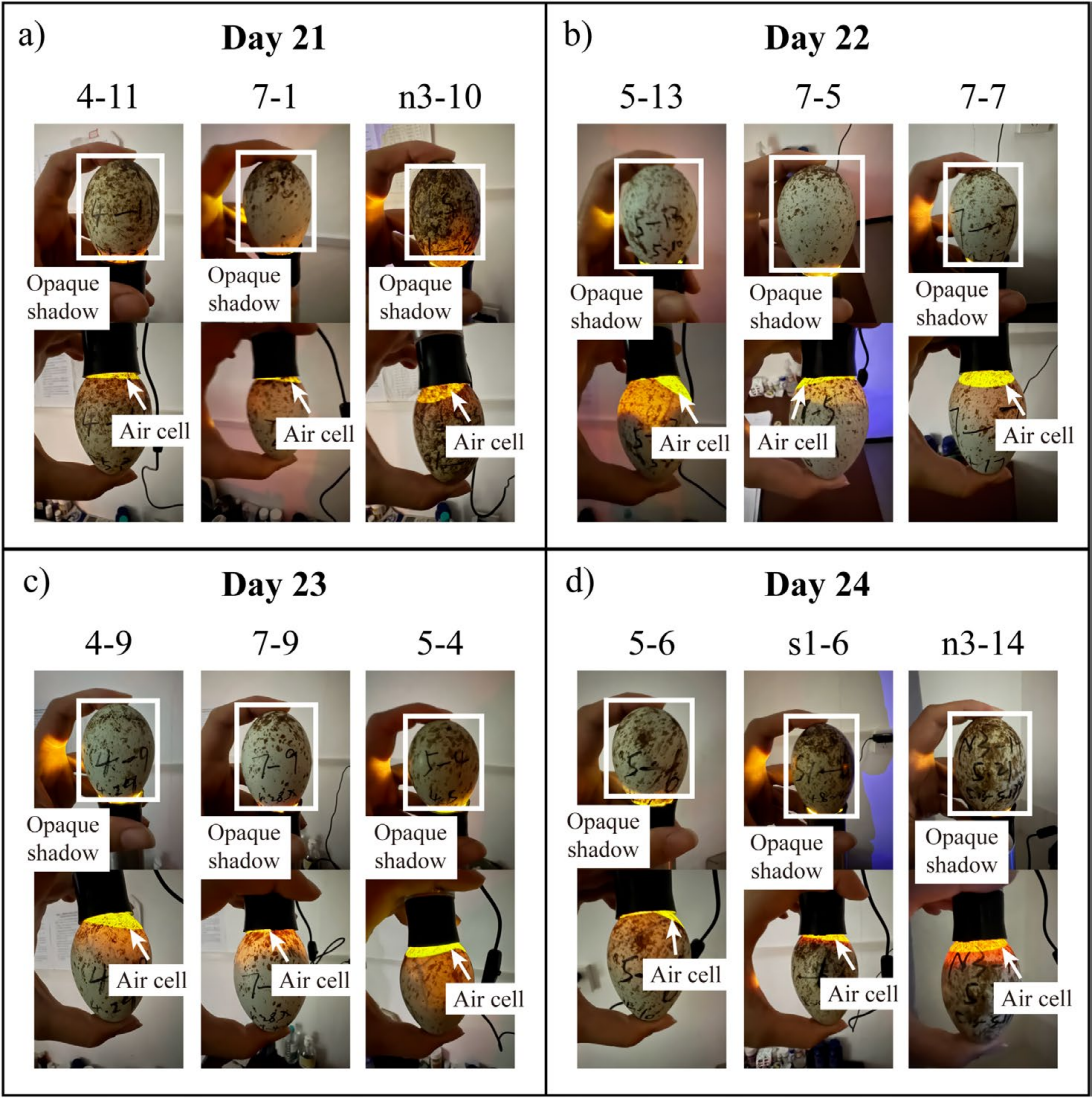
Representative candling images of fertile eggs during Day 21 and Day 24 of development. The shadows at the blunt end were highlighted in white boxes. The air cells were pointed out by white arrows. For each egg (4‐11, 7‐1, n3‐10, 5‐13, 7‐5, 7‐7, 4‐9, 7‐9, 5‐4, 5‐6, s1‐6, n3‐14), the images on the top were taken when candling from the sharp end, and those on the bottom were taken when candling from the blunt end.

Vascular structures begin to regress as the embryo enlarges. When candled from the sharp end, the shadow at the blunt end becomes nearly opaque, occupying almost the entire egg and greatly limiting light penetration (Figure 8a–d, white boxes). When candled from the blunt end, the air cell continues to enlarge gradually with smooth and sharply defined boundaries (Figure 8a–d, white arrows).

#### 3.2.7 Day 25–29

**FIGURE 9:**
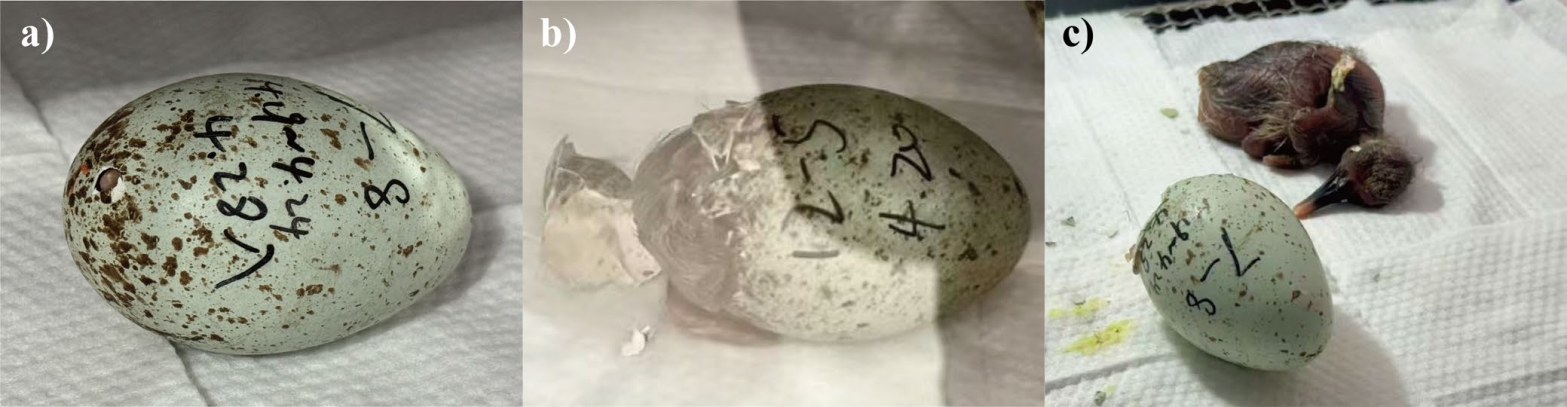
Hatching eggs and nestling that had just hatched out. (a) Egg 7‐8 on Day 26 when the bird started pipping; (b) Egg 12‐5 on Day 28 when the blunt end of the egg had been opened; (c) Nestling 7‐8 on Day 27 when it had just hatched out.

The embryo fills nearly the entire egg except for the air cell, which remains relatively stable in size with clear and well‐defined margins. Pipping and hatching occur during this stage (Figure 9).

### 3.3 Identification of Nonviable Eggs

By comparing the candling images throughout incubation, we were able to distinguish viable and nonviable eggs (Figure S1) and remove the latter timely before they decayed and caused contamination. In the following sections, we present some representative images and provide the detailed descriptions that help differentiate between the various types of eggs.

#### 3.3.1 Fertile Eggs Versus Infertile Eggs

**FIGURE 10:**
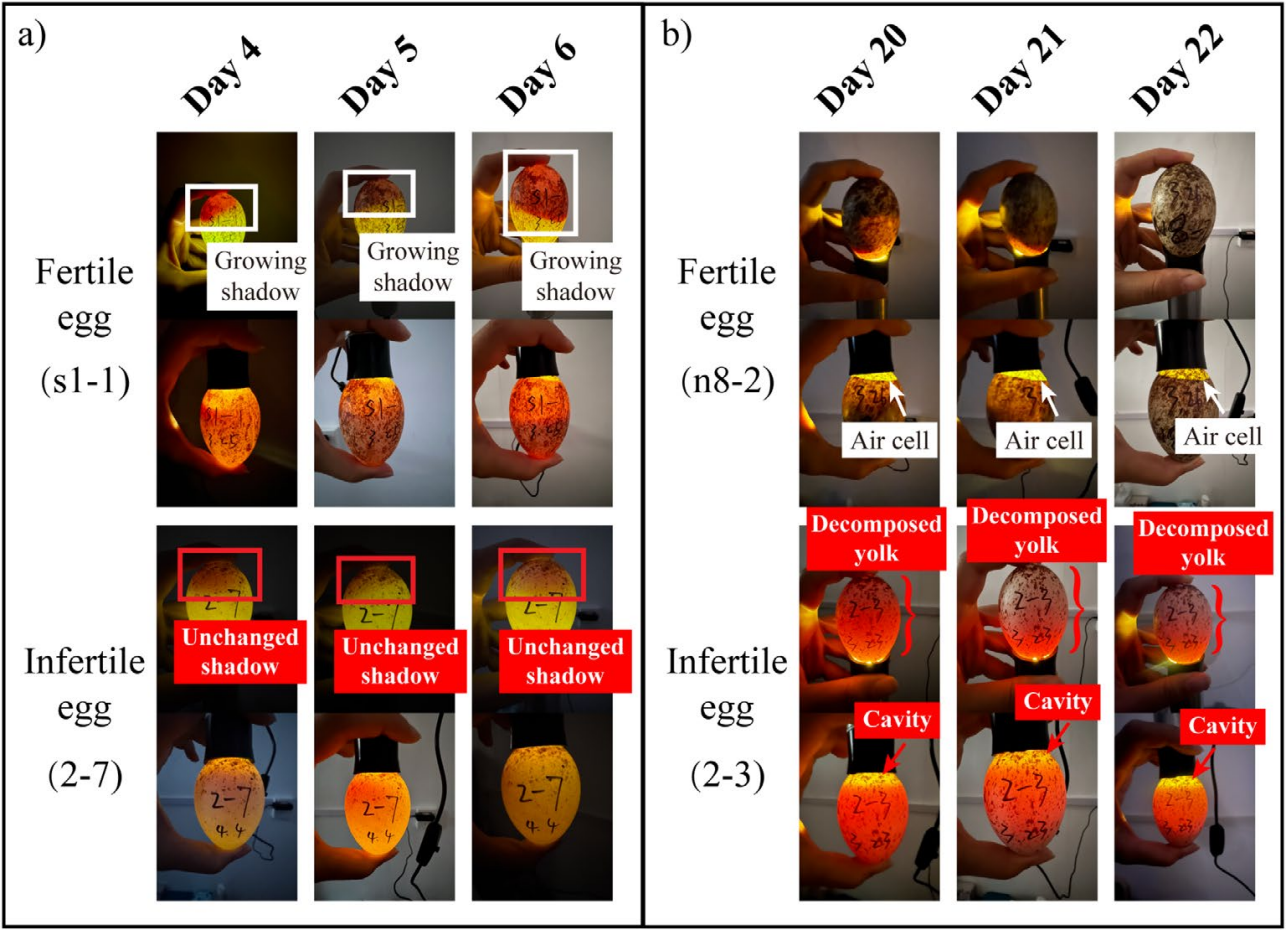
Representative candling images of fertile eggs (s1‐1, n8‐2) and infertile eggs (2‐7, 2‐3). (a) Images taken during Day 4 and Day 6 of development, when the growing shadow of embryo (highlighted with white boxes) inside fertile egg became visible, while the infertile egg showed no significant changes (highlighted with red boxes); (b) Images taken during Day 20 and Day 22 of development. In fertile eggs, the embryo's shadow almost filled the entire egg except the air cell (pointed out with white arrow), whereas in infertile egg, the yolk had decomposed and formed a reddish shadow (pointed out with red braces). Meanwhile, the cavity (pointed out with red arrow) in blunt end lacks clear boundaries. For each egg (s1‐1, 2‐7, n8‐2, 2‐3), the images on the top were taken when candling from the sharp end, and those on the bottom were taken when candling from the blunt end.

With continuous candling observations, fertile and infertile eggs can generally be distinguished during Day 4–6 of development (Figure 10a). When candled from the sharp end, the fertile eggs exhibit a progressively enlarging shadow caused by the developing embryo (Figure 8a, white boxes), whereas the shadow in the infertile eggs shows little change (Figure 10a, red boxes). When candled from the blunt end, the shadow in the infertile eggs remains roughly spherical rather than occupying a defined area. In some cases, particularly in the later stage of incubation, the shadow in infertile eggs also appears to occupy a certain area (Figure 10b, red braces). However, these eggs lack clearly defined air cells with distinct edges, which are characteristic of fertile eggs (Figure 10b, white arrows), but only a vague cavity (Figure 10b, red arrows).

#### 3.3.2 Embryonic Shadow Versus Decayed Yolk Shadow

**FIGURE 11:**
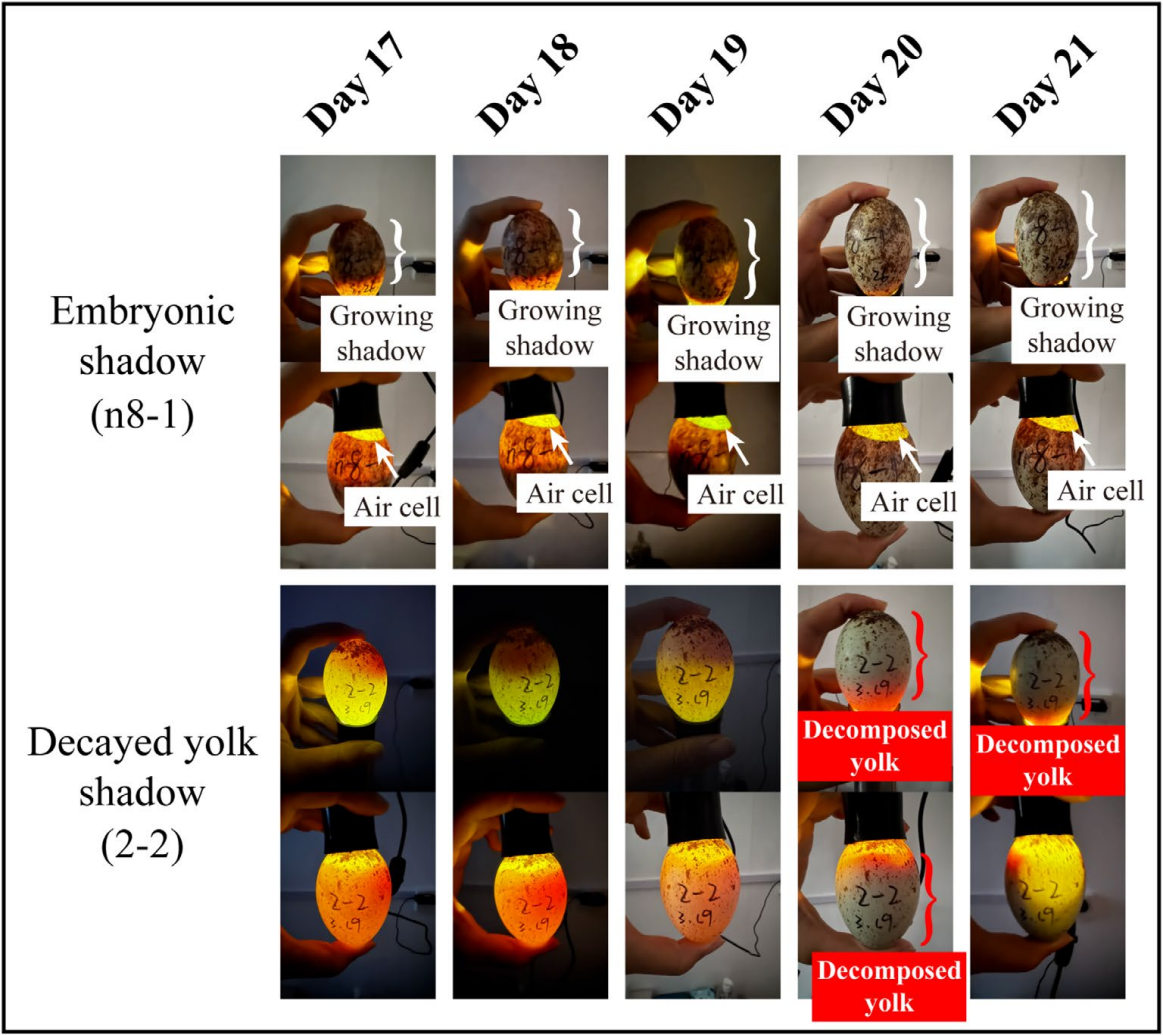
Representative candling images of fertile egg (n8‐1) and infertile egg (2‐2) during Day 17 to Day 21 of development. The growing shadow of embryo was highlighted with white braces and the air cell was pointed out by white arrow. The shadow of decomposed yolk without clear structure and boundaries was highlighted with red braces. For each egg (n8‐1, 2‐2), the images on the top were taken when candling from the sharp end, and those on the bottom were taken when candling from the blunt end.

When the eggs are not removed immediately after laying and continuous images of candling are unavailable, it may be difficult to distinguish fertilized from unfertilized eggs solely based on the presence of large shadowed areas. In fertilized eggs, the shadow gradually expands (Figure 11, white braces) as embryonic development progresses with a clear air cell that remains at the blunt end (Figure 11, white arrows). During the mid‐development stage, the embryo and its vascular network can be visualized under candling (Figures 6 and 7). In contrast, the extensive shadow observed in unfertilized eggs usually results from yolk decomposition and rupture, which mixes with the albumen. It typically appears abruptly within 1 or 2 days (Figure 11, images of egg 2‐2 on Day 19 and Day 20), with indistinct edges, a relatively uniform appearance, and no observable structural features (Figure 11, red braces).

#### 3.3.3 Living Embryo Versus Dead Embryo

**FIGURE 12:**
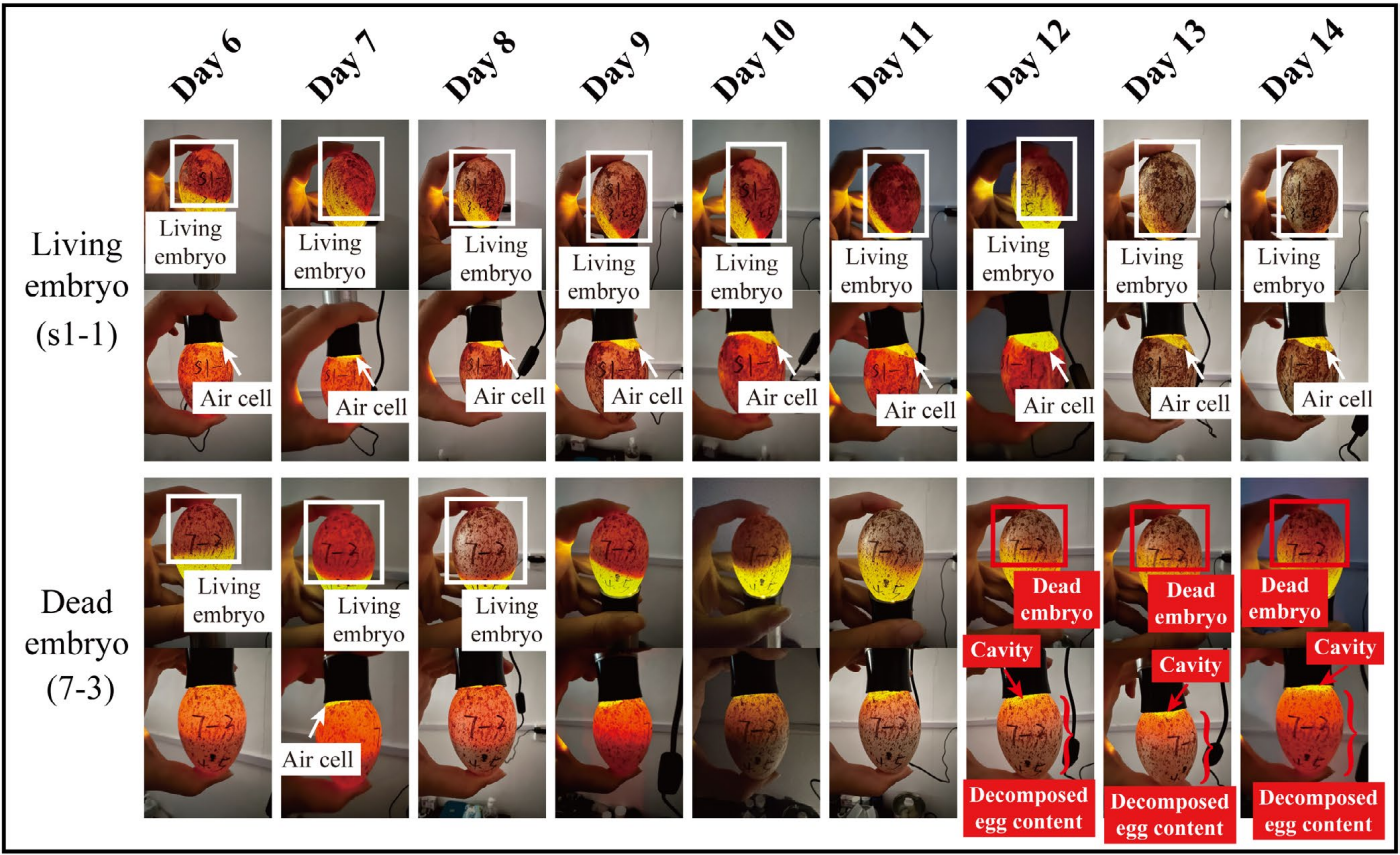
Representative candling images of living embryo (s1‐1) and dead embryo (7‐3, which died at around Day 10) during Day 6 to Day 14 of development. The living embryo was highlighted with white boxes, and the air cell was pointed out with white arrows. The dead embryo was highlighted with red boxes, and the shadow of decomposed egg content was pointed out with red braces. Meanwhile, the cavity (pointed out with red arrow) in blunt end lacks clear boundaries. For each egg (s1‐1, 7‐3), the images on the top were taken when candling from the sharp end, and those on the bottom were taken when candling from the blunt end.

Apart from differences in growth, the shadows of living and dead embryos exhibit other distinctive features. Living embryos display stable shadows and do not move even when the eggs are shaken slightly. The edges of both the shadow (Figure 12, white boxes) and the air cell are well‐defined (Figure 12, white arrows), and occasional embryonic movements may be observed (Figure 6, light blue arrow). In contrast, dead embryos show shifting shadows, the edges of which display diffuse red structures resulting from ruptured blood vessels (Figure 12, red boxes), and the borders of the air cell are blurred (Figure 12, red arrows). When the egg is tilted, the fluid appears to invade the area of the air cell, indicating disrupted internal structures. As the vessels or yolk sac ruptures, the egg contents become turbid, and the candling image may resemble that of an infertile egg with a decomposed yolk (Figures 11 and 12, red braces).

### 3.4 Validation of Diagnostic Features

#### 3.4.1 Classification of Developmental Stage

The observer accuracies were moderate (observer 1: 0.404; observer 2: 0.386; observer 3: 0.421; mean = 0.404), with low and consistent mean absolute error (observer 1: 0.754; observer 2: 0.754; observer 3: 0.737; mean = 0.749), indicating relatively small deviations from the true stages. Weighted Cohen's kappa showed that agreement between observer classifications and true stages was substantial for all observers (observer 1: κ = 0.723, observer 2: κ = 0.770, observer 3: κ = 0.774). Overall interobserver agreement was moderate (Krippendorff's α = 0.685), with pairwise weighted Cohen's kappa ranging from 0.649 to 0.815 (observer 1 vs. 2: κ = 0.649; observer 1 vs. 3: κ = 0.681; observer 2 vs. 3: κ = 0.815).

#### 3.4.2 Identification of Nonviable Eggs

For Day 4–6, observer accuracies in classifying fertilization status were high (observer 1: 0.949; observer 2: 0.923; observer 3: 0.846; mean = 0.906) with strong diagnostic performance (Table 2). Interobserver agreement was substantial, with an overall substantial Fleiss' kappa of 0.812 and pairwise Cohen's kappa ranging from 0.727 to 0.941 (observer 1 vs. 2: κ = 0.941; observer 1 vs. 3: κ = 0.780; observer 2 vs. 3: κ = 0.727).

**TABLE 2:** Diagnostic performance of observers in classifying fertilization status and embryo viability from candling images at different incubation stages.

Incubation process	Type	Observer	Sensitivity	Specificity	Precision	TP	TN	FP	FN
Day 4–6	Fertilization status	obs1	1	0.857	0.926	25	12	2	0
		obs2	0.96	0.857	0.923	24	12	2	1
		obs3	0.84	0.857	0.913	21	12	2	4
Day 6–24	Fertilization status	obs1	0.947	0.74	0.9	108	63	12	6
		obs2	0.991	0.667	0.819	113	50	25	1
		obs3	0.982	0.773	0.868	112	58	17	2
	Embryo viability	obs1	0.824	0.609	0.824	70	14	9	15
		obs2	0.767	0.926	0.767	66	25	2	20
		obs3	1	0.52	1	87	13	12	0

For Day 6–24, accuracies in classifying fertilization status remained high (observer 1: 0.905; observer 2: 0.862; observer 3: 0.888; mean = 0.889). Sensitivity was consistently high, whereas specificity showed greater variation and precision remained relatively high, suggesting strong overall diagnostic performance (Table 2). Interobserver agreement remained substantial, with an overall Fleiss' kappa of 0.813 and Cohen's kappa ranging from 0.758 to 0.860 (observer 1 vs. 2: κ = 0.758; observer 1 vs. 3: κ = 0.824; observer 2 vs. 3: κ = 0.86). As for classification of embryo viability, the accuracies were relatively lower (observer 1: 0.778; observer 2: 0.805; observer 3: 0.893; mean = 0.825), and diagnostic performance varied among observers (Table 2). Interobserver agreement was relatively low, with an overall Fleiss' kappa of 0.274 and pairwise Cohen's kappa ranging from 0.291 to 0.349 (observer 1 vs. 2: κ = 0.291; observer 1 vs. 3: κ = 0.349; observer 2 vs. 3: κ = 0.347).

Regarding the relationship between diagnostic features and observed egg outcomes, several features were identified as significant predictors of fertilization status and embryo viability (Table 3). For Day 4–6, fertilization status was significantly positively associated with the presence of distinct shadow edge when candled from the blunt end (*b_shadow_with_distinct_edge*). For Day 6–24, several features were found positively associated with fertilization status. The broad shadows when candled from the sharp and blunt end (*s_broad_shadow, b_broad_shadow*) were both significant predictors. Additional significant positive effects were detected for shadow coverage exceeding 50% when candled from the sharp end (*s_shadow_coverage_gt50*), as well as distinct shadow edge (*b_shadow_with_distinct_edge*), vascular structures (*b_vascular_structure*), and air cell with clear boundary (*b_aircell_with_distinct_edge*) when candled from the blunt end. As for embryo viability, the presence of distinct shadow edge (*s_shadow_with_distinct_edge*) and a uniform dark shadow (*s_uniform_dark_shadow*) when candled from the sharp end, as well as an air cell with clear boundary when candled from the blunt end (*b_aircell_with_distinct_edge*), were positively associated with viable embryos, whereas other features showed no significant associations.

**TABLE 3:** Associations between diagnostic features and fertilization status, as well as embryo viability, across incubation stages.

Incubation process	Type	Diagnostic features	Estimate ± SE	*z*	*p*
Day 4–6	Fertilization status	Intercept	−2.378 ± 0.806	−2.951	—
		s_broad_shadow	−0.023 ± 1.142	−0.02	0.984
		s_shadow_with_distinct_edge	1.369 ± 0.731	1.873	0.061
		b_broad_shadow	1.342 ± 0.876	1.532	0.126
		b_shadow_with_distinct_edge	2.605 ± 0.676	3.856	**< 0.001**
Day 6–24	Fertilization status	Intercept	−4.620 ± 0.802	−5.758	—
		s_broad_shadow	2.542 ± 0.825	3.082	**0.002**
		s_shadow_with_distinct_edge	0.199 ± 0.415	0.481	0.631
		s_shadow_coverage_gt50	0.789 ± 0.356	2.217	**0.027**
		s_uniform_dark_shadow	−0.156 ± 0.386	−0.403	0.687
		s_vascular_structures	−0.151 ± 0.799	−0.189	0.850
		b_broad_shadow	1.532 ± 0.479	3.198	**0.001**
		b_shadow_with_distinct_edge	0.857 ± 0.398	2.155	**0.031**
		b_vascular_structures	1.627 ± 0.763	2.132	**0.033**
		b_aircell_with_distinct_edge	1.793 ± 0.389	4.614	**< 0.001**
	Embryo viability	Intercept	−2.061 ± 0.591	−3.489	—
		s_shadow_with_distinct_edge	1.342 ± 0.421	3.191	**0.001**
		s_shadow_coverage_gt50	0.302 ± 0.574	0.526	0.599
		s_uniform_dark_shadow	0.894 ± 0.409	2.184	**0.029**
		s_vascular_structures	0.101 ± 0.603	0.167	0.867
		b_shadow_with_distinct_edge	−0.234 ± 0.417	−0.561	0.575
		b_vascular_structures	0.634 ± 0.533	1.190	0.234
		b_aircell_with_distinct_edge	2.166 ± 0.387	5.598	**< 0.001**

### 3.5 Critical Periods in Embryo Development

Based on four embryos with recorded death times (5‐9, 4‐6, n4‐5, n3‐11), developmental stages could be partly characterized as follows: Day 7, the embryo was < 2 cm, with distinct eye pigmentation, rudimentary limb buds, and visible heart chambers; by Day 9, the eyes had enlarged and the limbs were more prominent; by Day 15, the beak was clearly developed, the head was nearly equal in size to the body with large and prominent eyes, and the limbs were noticeably more advanced; by Day 23, feathers were already developed, the eyes were covered by eyelids, limb structures were fully visible, the beak had hardened, and egg teeth had appeared. Additionally, 3 embryos that died due to abnormal fetal position (n8‐1, 5‐4, 15‐3) had their head and beak compressed or blocked by other body parts, preventing successful pipping (Figure S1).

**FIGURE 13:**
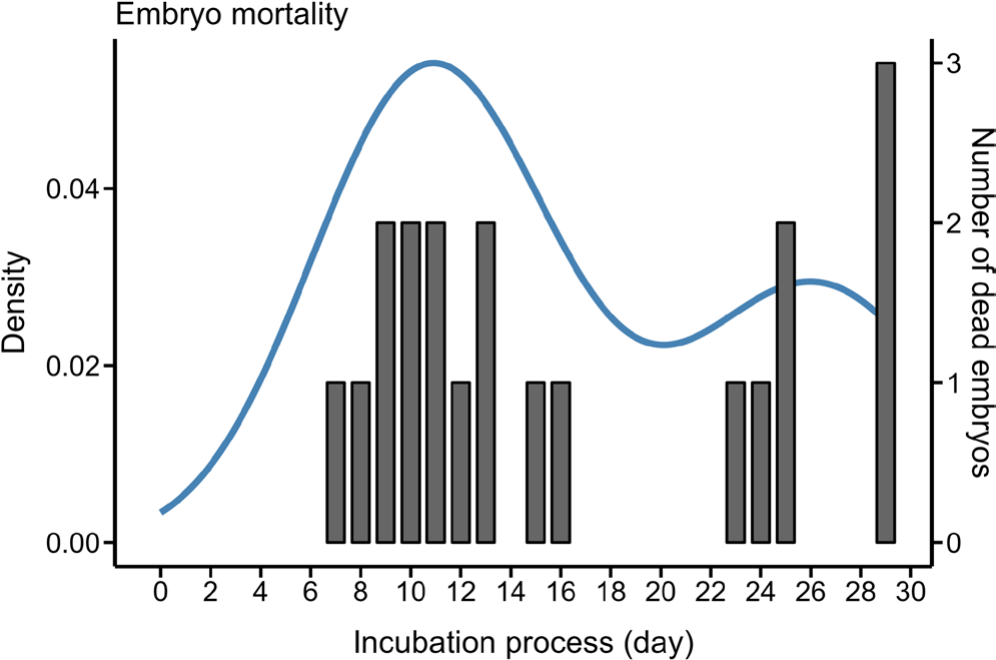
Estimated times of death for dead embryos (excluding 3 that died from accidents and 2 for which no egg‐opening images were available). The left vertical axis represents the density distribution of dead embryos across different incubation ages, while the right vertical axis indicates the exact number of dead embryos recorded on each day.

Using these four reference embryos and the summarized differences in candling images between living and dead embryos, we estimated the death times for other 20 embryos (excluding 3 that died from accidents and 2 for which no egg‐opening images were available) and placed them on the developmental timeline. Embryo mortality was found to be concentrated during the mid‐stage of incubation (Days 7–15) (*N* = 12, 60.0%) and near hatching (Days 23–29) (*N* = 7, 35.0%) (Figure 13), suggesting that these critical periods in the development of crested ibis embryos require particular attention.

## 4 Discussion

In this study, we present the first comprehensive description of egg development, encompassing both the changes observed in unfertilized eggs and daily embryonic development in the endangered crested ibis based on candling images throughout incubation (Figures 3, 4, 5, 6, 7, 8, 9). By summarizing characteristic features at different developmental stages, we provide a practical reference for assessing fertilization status and embryo viability (Figures 10, 11, 12), which can facilitate the early detection of abnormalities and timely management interventions. In addition, by integrating morphological characteristics with candling records, we estimated the timing of embryo mortality and found that most deaths occurred during mid‐incubation (Day 7–15) (*N* = 12, 60.0%) and shortly before hatching (Day 23–29) (*N* = 7, 35.0%) (Figure 13, Figure S1). These findings reveal critical periods in the embryonic development of crested ibis that deserve particular attention and provide an important basis for improving hatching success and understanding developmental constraints in this endangered species.

By comparing all the candling images, we found that, in general, the differences in shadow between infertile and fertilized eggs became discernible after approximately 4–6 days of incubation (Figure 4). This pattern is consistent with our validation results, which showed that fertilization status could be identified with high accuracy at this stage. In particular, the presence of distinct shadow edge when candled from the blunt end provided strong discriminatory power. This likely reflects early structural differences between fertilized and unfertilized eggs. Based on the morphological information from other avian species with comparable incubation periods (~28 days), the embryo at this stage shows a complete flexure from head to tail, with increasingly developed vascularization, and the early differentiation of major organs and body structures including the eyes, heart, and limbs (Fant 1957; Caldwell and Snart 1974; de Araújo et al. 2019; Pisenti et al. 2024). These developmental changes likely contribute to the formation of distinct shadows in fertilized eggs that are absent in infertile eggs. However, according to the Hamburger & Hamilton stages of development and studies of species with similar incubation periods, the actual onset of embryonic development occurs earlier (Hamburger and Hamilton 1951; Caldwell and Snart 1974; de Araújo et al. 2019; Pisenti et al. 2024), but it can only be directly confirmed through a timely breakout examination. Regarding the features previously reported in studies on chickens that can be used to identify nonviable eggs, the typical indicator of early embryonic death is the appearance of a “blood ring” when inspecting the eggs (Ernst et al. 2004). This disorder has been described as an autosomal recessive lethal trait (Savage et al. 1988). In our study, we did not observe such a distinct “blood ring” when candling. This may be attributed to the relatively dark coloration and low translucency of the eggshell, as well as the presence of dark spots on the surface, which may hinder observation. Interspecific differences in the manifestation of embryo death might also account for this discrepancy.

At later stages (Day 6–24), fertilization assessment relied on a broader combination of features, including shadow extent, vascularization, and air cell characteristics, suggesting that multiple visual cues emerge as embryonic development progresses. Assessment of embryo viability was comparatively more challenging, with lower accuracy and interobserver agreement, but still demonstrated reliable identification, indicating that these features hold valuable potential for practical applications. When embryos die after a period of development, the major indicators of death are typically a large, dark, round shadow and the absence of visible blood vessels (Ernst et al. 2004). This pattern is consistent with our observations in crested ibis eggs that experienced embryonic death, where vascular structures disappeared and the edges of the shadow became blurred after death (Figure 12). Validation of diagnostic features showed that a uniform dark shadow with distinct edges when candled from the sharp end is a reliable indicator of embryo survival, while the presence of vascular structures did not significantly contribute to the identification of embryo viability. This may be due to limited image quality and interference from the eggshell surface, which can make vascular features difficult to detect.

Based on the egg‐opening images of four embryos with recorded death times, we estimated the timing of death for most embryos by comparing their images taken during incubation and after egg opening. Our results indicated that embryo mortality primarily occurred during the mid‐incubation stage (Day 7–15) and near hatching (Day 23–29), indicating that these may represent critical periods in embryonic development. Previous studies have reported that avian embryos are more likely to die during the early and late stages of incubation (Romanoff 1949). Research on a threatened bird, the Hihi ( *Notiomystis cincta* ), revealed that early embryo mortality (Day 0–5) accounted for an average of 56.8% (±15% SD) of total embryo deaths (Morland et al. 2024). In the study conducted by Yang et al. (2020) on the artificial breeding of crested ibis in Sichuan, China (2017–2019), the highest mortality occurred during Day 17–25 (57.7%–75.8%), followed by Day 9–16 (9.1%–34.7%). The differences in the conclusions of different studies may be related to interspecies differences, the different division of the incubation stage, and the differences in the incubation environment. Common nongenetic causes of embryo death include nutrient deficiencies in the eggs, bacterial contamination, abrupt changes in temperature or humidity, inadequate or improper turning, and so on (Wilson 1997; Ori 2011; Rideout 2012). In the current artificial breeding process of crested ibis, procedures such as manual egg collection and cooling, as well as microenvironmental differences among incubators, could contribute to embryo mortality. Therefore, identifying the specific causes of hatching failure based on assessments of egg condition and developmental stage would facilitate targeted improvements in management practices. For example, operations such as transferring eggs from natural to artificial incubation or changing incubators during critical periods are best avoided, as these may introduce mechanical shock or prolonged cooling, potentially compromising embryo survival. Additionally, previous studies have shown that deficiencies in specific nutrients can increase embryonic mortality at particular stages in chickens (Romanoff 1949). Thus, identifying when embryos die may provide indirect insights into possible nutritional limitations, to inform targeted dietary supplementation for breeding adults to improve egg quality and embryo survival.

Additionally, we noticed that three embryos (Figure S1, n8‐2, 5‐4 and 15‐3) that had fully developed and exhibited pre‐hatching behaviors, such as chirping and shaking, ultimately failed to hatch and died. Dissection revealed that their heads and beaks were obstructed by other body parts, preventing successful pipping (Figure S1). During the late stage of incubation, embryos rotate to position themselves for hatching, and factors such as rough handling and improper turning can disrupt this process (Rideout 2012). Small egg size may also be a contributing factor, as limited internal space can restrict the embryo's movement. In our observations, egg 15‐3, which was produced by a pair of 1‐year‐old birds, was notably small, measuring only 62.92 mm in length and 40.78 mm in width—both shorter and narrower than the average dimensions of normally hatched eggs (66.59 [62.4–71.28] mm and 45.92 [41.3–48.5] mm). Reported length and width in previous studies were approximately 67.55 and 45.24 mm (Shi et al. 1989; Xi et al. 2001). The confined space likely restricted the embryo's movement, hindering the rotational behavior necessary for pipping. Therefore, close monitoring is required during the late stages of incubation, particularly as embryos approach hatching. Signs of unsuccessful pipping, such as prolonged pre‐hatching activity without shell breakthrough, should be carefully assessed. When necessary, assisted hatching through careful manual intervention (e.g., partial shell removal) may help prevent mortality, provided that such procedures should be conducted cautiously to avoid injury to the embryo.

Although the current population of the crested ibis has greatly increased, the species still faces severe inbreeding and low genetic diversity, as the entire existing population originated from only seven individuals and two breeding pairs. Previous research showed that the nucleotide diversity of existing populations of the crested ibis is markedly lower than that of the historic population, and a substantial accumulation of deleterious mutations has occurred over the past four decades, posing potential genetic risks (Feng et al. 2019). Additionally, inbreeding in crested ibis has already been found to be associated with increased embryonic mortality, and several candidate genes linked to detrimental diseases were identified in genomic regions that differ between viable and dead embryo samples (Fu et al. 2019), suggesting potential impacts on embryo quality and increased sensitivity to environmental fluctuations during incubation. Still, we need a more precise understanding of which specific stages of embryonic development are affected. Our study has provided a comprehensive description of egg development based on candling images throughout incubation, allowing more precise determination of fertilization status and the timing of embryo mortality. These results laid a critical foundation for identifying the causes of embryo death and for explaining how inbreeding affects egg quality and embryo survival, particularly by revealing the developmental stages at which its effect may be most pronounced.

At present, most guidelines for identifying nonviable eggs through candling have been developed for economically important species such as chickens and turkeys, whereas comparable references for endangered species like crested ibis remain relatively limited. Previous studies on this species have largely focused on quantitative metrics such as clutch size, fertilization rate, and hatching rate, with relatively little attention to detailed observations and descriptions of the embryonic development process (Liu et al. 1999; Xi et al. 2001; Huang et al. 2006, 2016). Although candling has been applied in practical artificial breeding, the absence of standardized descriptions for candling results means that assessments of egg fertilization status and embryonic development often rely heavily on the experience and skill of individual staff, increasing the risk of inconsistency and misclassification. Furthermore, without systematic references, eggs are often incubated for extended periods to avoid prematurely terminating the incubation of fertilized eggs, which may result in dead embryos or infertile eggs deteriorating and increase contamination risks within incubators. Based on 1422 images from 98 eggs, we have preliminarily summarized the egg development patterns of crested ibis, providing detailed descriptions and illustrative references. This reference can offer practical guidance for researchers and breeding staff to accurately stage normal embryos and timely identify and remove nonviable eggs, thereby improving incubation management and reducing contamination risks. In addition, given the limited research on the embryonic development of the crested ibis and its close relatives, the developmental information generated in this study represents an important complement to existing research on avian embryogenesis. The diagnostic features documented here offer a valuable resource for future research, not only in the crested ibis, but also in closely related species and others with similar developmental patterns. This broadens the scope of avian developmental studies, which have previously focused mainly on economically important or model species, thus facilitating interspecies comparisons across a wider range of avian taxa.

Strictly speaking, a complete description of egg development should also include detailed morphological data obtained through direct examination of fertilized eggs, as has been conducted in previous studies on other avian species (Hamburger and Hamilton 1951; Sellier et al. 2006; Nagai et al. 2011; Hemmings and Birkhead 2016). However, due to conservation constraints, it is currently not feasible to acquire such detailed morphological data for the crested ibis through invasive methods like egg dissection. Meanwhile, in our study, the examination of infertile eggs and those containing dead embryos was conducted only after confirming that there were no signs of life. By that time, most eggs had already begun to decay, and many embryonic structures were no longer clearly distinguishable, which inevitably limited the accuracy of our descriptions and hindered direct comparison with classical embryological staging systems (e.g., Hamburger and Hamilton 1951) that rely on detailed morphological characterization. This may also have resulted in some fertilized eggs that died at very early stages (Day 0–3) being misclassified as unfertilized eggs due to the lack of visible embryonic features in the candling images, thereby leading to a possible overestimation of the infertility rate and an underestimation of early embryonic mortality. Therefore, improving noninvasive observation protocols and the quality of images, enabling timely identification of nonviable eggs followed by standardized breakout examination, and continuing to accumulate data will be essential to refine our understanding of crested ibis egg development. Only through detailed documentation and quantitative validation of both normal developmental stages and the characteristics of embryos in different conditions can we gain a comprehensive understanding of developmental patterns, which will further improve their applicability in practical management and provide a stronger basis for future comparative analyses across species.

## 5 Conclusion

We present the first description of egg development in the crested ibis based on candling images throughout incubation. Our study also provides a practical guideline for assessing fertilization status and embryo viability, including indicators such as the growth rate and shape of the shadow, and the presence of clearly defined air chambers, which can facilitate the early detection of abnormalities and enable timely management interventions. Furthermore, by integrating morphological characteristics with candling records, we estimated the timing of embryo mortality and found that most deaths occurred during mid‐incubation (Day 7–15) and near hatching (Day 23–29), which reveals the critical periods of embryonic development that warrant particular attention. Overall, our findings offer an important basis for improving hatching success and enhancing understanding of embryo developmental constraints in this endangered species, which also provide an important addition to existing studies of avian embryonic development and lays a foundation for future comparative analyses across species.

## Author Contributions

**Yuansi He:** data curation (lead), formal analysis (lead), investigation (lead), methodology (equal), project administration (equal), validation (equal), visualization (lead), writing – original draft (lead), writing – review and editing (equal). **Xuebo Xi:** data curation (equal), formal analysis (equal), investigation (equal), writing – review and editing (equal). **Siyi Zeng:** data curation (equal), formal analysis (supporting), investigation (equal), writing – review and editing (supporting). **Hua Huang:** data curation (equal), investigation (equal), resources (equal). **Xiangjiang Zhan:** conceptualization (equal), funding acquisition (equal), project administration (equal), resources (equal), supervision (lead), validation (lead), writing – review and editing (equal). **Daiping Wang:** conceptualization (lead), data curation (equal), formal analysis (equal), funding acquisition (equal), investigation (equal), methodology (lead), project administration (lead), resources (equal), supervision (lead), validation (lead), visualization (equal), writing – original draft (supporting), writing – review and editing (equal).

## Funding

The research is funded by the National Natural Science Foundation of China (General Program, Grant No. 32570596, 32125005); Initiative Scientific Research Program, Institute of Zoology, CAS (2023IOZ0104, 2024IOZ0107); Joint Research Unit of Chinese Academy of Sciences (JRU CAS:152111ZYLH20250004); The International Partnership Program of the Chinese Academy of Sciences for Grand Challenges (073GJHZ2023091GC); The National Key Program of Research and Development, Ministry of Science and Technology of China (2023YFF1304800).

## Ethics Statement

All examination procedures were under the guidance of the Ethics Committee of the Institute of Zoology, Chinese Academy of Sciences. The collection and processing of Crested ibis tissues in this study were conducted in accordance with the guidelines of Institutional Anima Care and Use Committee of the Institute of Zoology, Chinese Academy of Sciences.

## Consent

The authors have nothing to report.

## Conflicts of Interest

The authors declare no conflicts of interest.

## Supporting information


**Table S1:** Summary of the number of eggs and images recorded throughout the incubation. Day 0 referred to the day when the egg was laid. For eggs in which the embryos eventually died, images taken before any visible signs of abnormality appeared were classified as normal embryo.
**Figure S1:** The dead embryos of the Dongzhai Crested ibis population in 2025 breeding season with images after breakout examination. Egg 5‐9, 4‐6, n4‐5, and n3‐11 died in accidents with documented death times. Egg n8‐1, 5‐4, and 15‐3 died from abnormal fetal position. The times of death for other embryos were estimated based on their candling images and the morphological features.

## Data Availability

The data is available on: https://doi.org/10.5061/dryad.fbg79cp9z.
