# Supplementary material for: Candling Analysis of Egg Development in an Endangered Bird Species Crested Ibis ( Nipponia nippon )
Source: Ecol Evol. 2026 Jun 16;16(6):e73797. doi: 10.1002/ece3.73797 (PMC13270401; doi:10.1002/ece3.73797)
Supplement: Supplementary file 1 — Table S1: Summary of the number of eggs and images recorded throughout the incubation. Day 0 referred to the day when the egg was laid. For eggs in which the embryos eventually died, images taken before any visible signs of abnormality appeared were classified as normal embryo. Figure S1: The dead embryos of the Dongzhai Crested ibis population in 2025 breeding season with images after breakout examination. Egg 5‐9, 4‐6, n4‐5, and n3‐11 died in accidents with documented death times. Egg n8‐1, 5‐4, and 15‐3 died from abnormal fetal position. The times of death for other embryos were estimated based on their candling images and the morphological features. [file ECE3-16-e73797-s001.zip › Candling analysis_sup.docx]

# Candling analysis of egg development in an endangered bird species crested ibis (*Nipponia nippon*)

Table S1. Summary of the number of eggs and images recorded throughout the incubation. Day 0 referred to the day when the egg was laid. For eggs in which the embryos eventually died, images taken before any visible signs of abnormality appeared were classified as **normal embryo**.

| Incubation process | **Infertile** | | **Abnormal embryo** | | **Normal embryo** | |
| --- | --- | --- | --- | --- | --- | --- |
|  | Eggs | Images | Eggs | Images | Eggs | Images |
| Day 0 | 3 | 6 | 0 | 0 | 0 | 0 |
| Day 1 | 19 | 38 | 0 | 0 | 27 | 54 |
| Day 2 | 10 | 20 | 0 | 0 | 13 | 26 |
| Day 3 | 14 | 28 | 0 | 0 | 14 | 28 |
| Day 4 | 12 | 24 | 0 | 0 | 19 | 37 |
| Day 5 | 15 | 30 | 0 | 0 | 23 | 46 |
| Day 6 | 10 | 20 | 0 | 0 | 16 | 32 |
| Day 7 | 9 | 17 | 0 | 0 | 16 | 32 |
| Day 8 | 9 | 18 | 0 | 0 | 18 | 36 |
| Day 9 | 16 | 31 | 2 | 4 | 20 | 40 |
| Day 10 | 11 | 22 | 2 | 4 | 14 | 28 |
| Day 11 | 10 | 20 | 5 | 10 | 14 | 28 |
| Day 12 | 14 | 28 | 4 | 8 | 12 | 24 |
| Day 13 | 13 | 28 | 9 | 18 | 16 | 32 |
| Day 14 | 8 | 16 | 4 | 8 | 15 | 30 |
| Day 15 | 12 | 24 | 5 | 10 | 10 | 20 |
| Day 16 | 9 | 18 | 5 | 10 | 9 | 18 |
| Day 17 | 16 | 32 | 8 | 16 | 18 | 36 |
| Day 18 | 12 | 24 | 5 | 10 | 8 | 16 |
| Day 19 | 11 | 22 | 4 | 8 | 11 | 22 |
| Day 20 | 10 | 20 | 1 | 2 | 11 | 22 |
| Day 21 | 10 | 20 | 2 | 4 | 12 | 24 |
| Day 22 | 8 | 16 | 1 | 2 | 9 | 18 |
| Day 23 | 7 | 14 | 1 | 2 | 12 | 24 |
| Day 24 | 6 | 12 | 1 | 2 | 5 | 10 |
| Day 25 | 5 | 10 | 1 | 2 | 11 | 22 |
| Day 26 | 3 | 6 | 2 | 4 | 5 | 9 |
| Day 27 | 3 | 6 | 0 | 0 | 2 | 4 |
| Day 28 | 1 | 2 | 1 | 2 | 2 | 4 |
| Day 29 | 0 | 0 | 0 | 0 | 1 | 2 |
| Total |  | 572 |  | 126 |  | 724 |


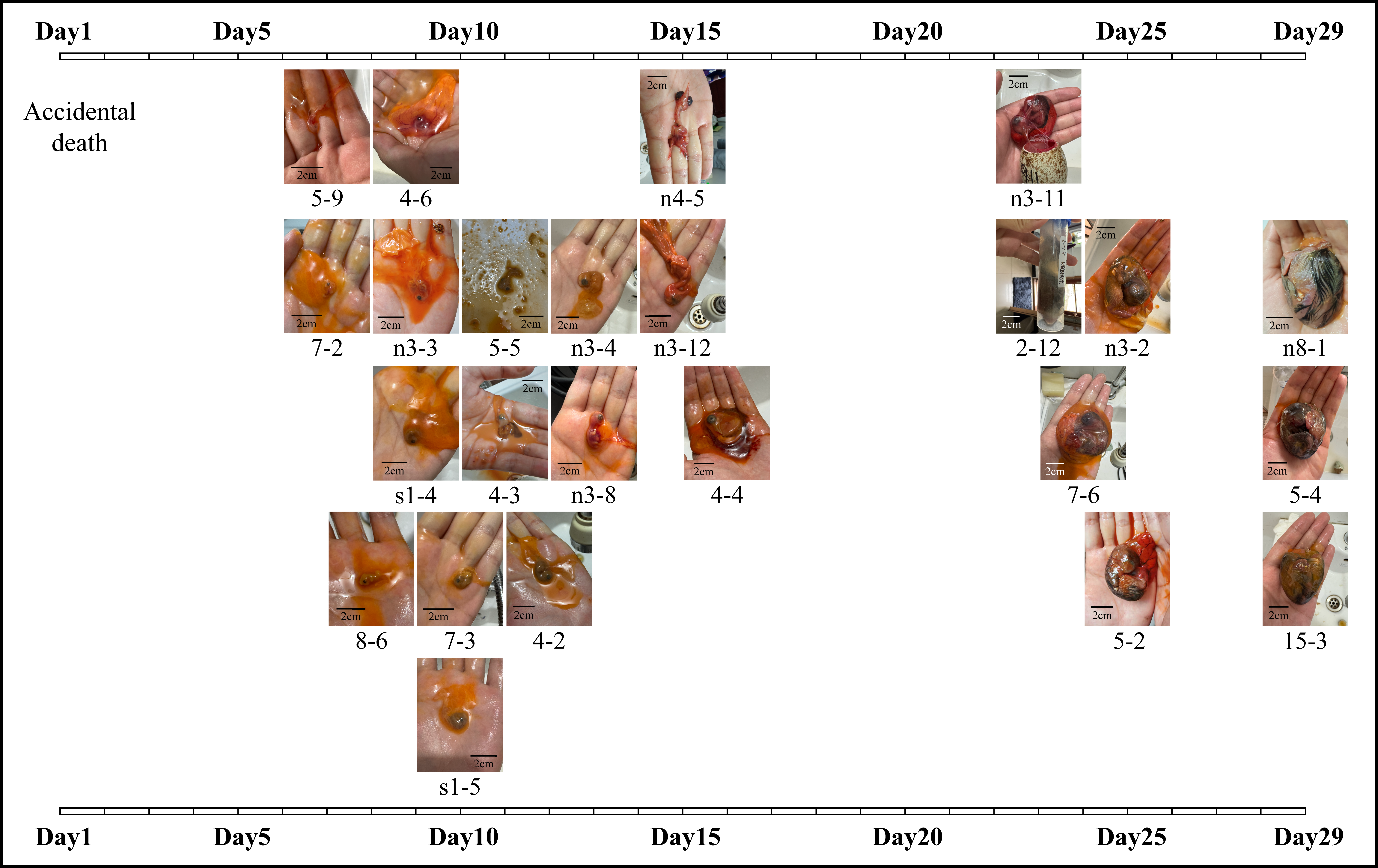
Figure S1. The dead embryos of the Dongzhai Crested ibis population in 2025 breeding season with images after break-out examination. Egg 5-9, 4-6, n4-5 and n3-11 died in accidents with documented death times. Egg n8-1, 5-4 and 15-3 died from abnormal fetal position. The times of death for other embryos were estimated based on their candling images and the morphological features.
