# Supplementary figures and images for: Candling Analysis of Egg Development in an Endangered Bird Species Crested Ibis ( Nipponia nippon )
Source: Ecol Evol. 2026 Jun 16;16(6):e73797. doi: 10.1002/ece3.73797 (PMC13270401; doi:10.1002/ece3.73797)

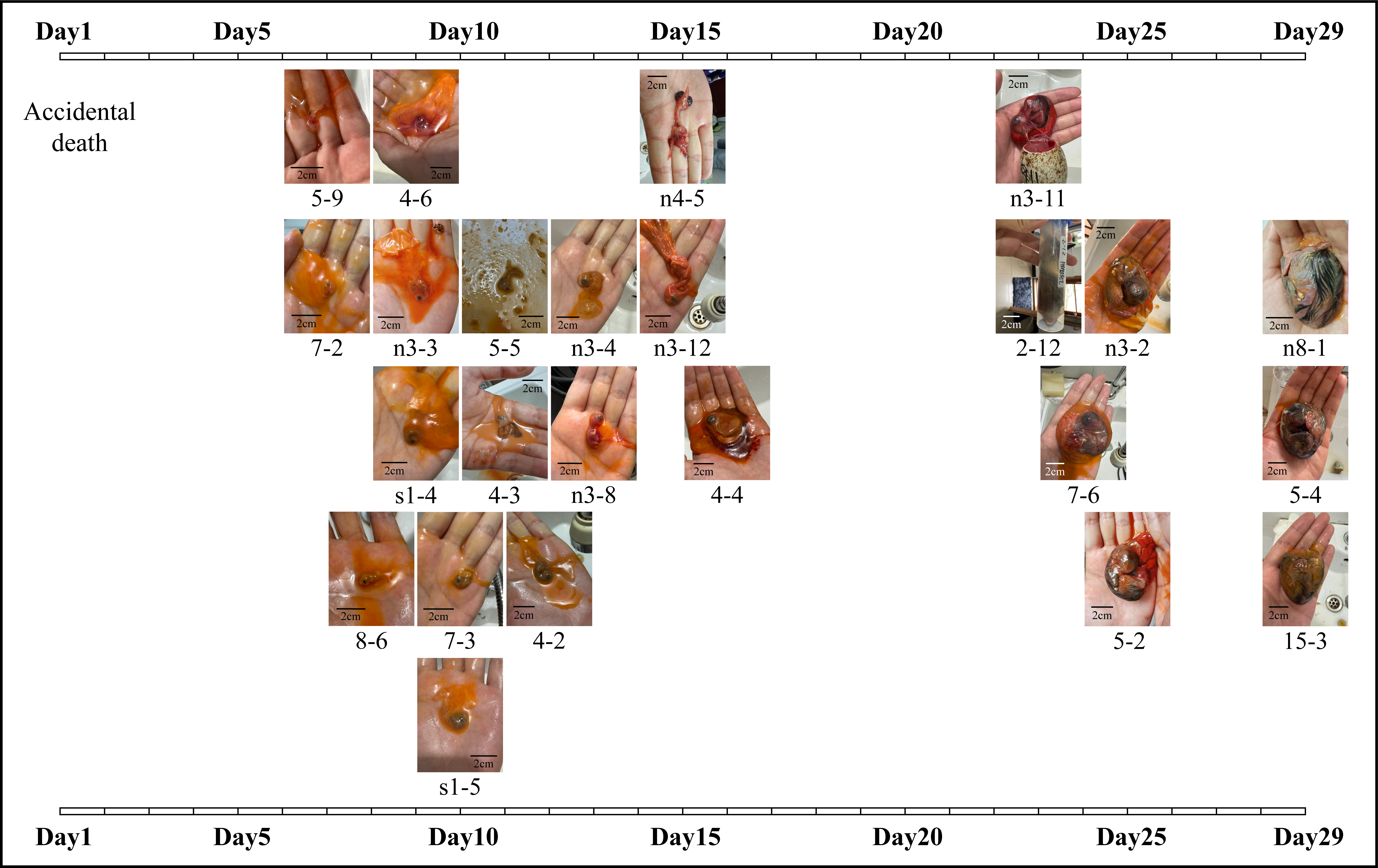

Supplement: Supplementary file 1 — Table S1: Summary of the number of eggs and images recorded throughout the incubation. Day 0 referred to the day when the egg was laid. For eggs in which the embryos eventually died, images taken before any visible signs of abnormality appeared were classified as normal embryo. Figure S1: The dead embryos of the Dongzhai Crested ibis population in 2025 breeding season with images after breakout examination. Egg 5‐9, 4‐6, n4‐5, and n3‐11 died in accidents with documented death times. Egg n8‐1, 5‐4, and 15‐3 died from abnormal fetal position. The times of death for other embryos were estimated based on their candling images and the morphological features. [file ECE3-16-e73797-s001.zip › Figure S1_breakout examination.png]
